# Extraintestinal Pathogenic *Escherichia coli* Utilizes Surface-Located Elongation Factor G to Acquire Iron from Holo-Transferrin

**DOI:** 10.1128/spectrum.01662-21

**Published:** 2022-03-07

**Authors:** Yu Sun, Xuhang Wang, Qianwen Gong, Jin Li, Haosheng Huang, Feng Xue, Jianjun Dai, Fang Tang

**Affiliations:** a MOE Joint International Research Laboratory of Animal Health and Food Safety, College of Veterinary Medicine, Nanjing Agricultural University, Nanjing, China; b Key Lab of Animal Bacteriology, Ministry of Agriculture, College of Veterinary Medicine, Nanjing Agricultural University, Nanjing, China; c School of Life Science and Technology, China Pharmaceutical University, Nanjing, China; Emory University School of Medicine

**Keywords:** extraintestinal pathogenic *Escherichia coli*, transferrin-binding protein, elongation factor G, iron acquisition, intracellular survival

## Abstract

Extraintestinal pathogenic Escherichia coli (ExPEC) can cause systemic infections in both humans and animals. As an essential nutrient, iron is strictly sequestered by the host. Circumventing iron sequestration is a determinant factor for ExPEC infection. However, the ExPEC iron acquisition mechanism, particularly the mechanism of transferrin (TF) acquisition, remains unclear. This study reports that iron-saturated holo-TF can be utilized by ExPEC to promote its growth in culture medium and survival in macrophages. ExPEC specifically bound to holo-TF instead of iron-free apo-TF via the surface located elongation factor G (EFG) in both culture medium and macrophages. As a moonlighting protein, EFG specifically bound holo-TF and also released iron in TF. These two functions were performed by different domains of EFG, in which the N-terminal domains were responsible for holo-TF binding and the C-terminal domains were responsible for iron release. The functions of EFG and its domains have also been further confirmed by surface-display vectors. The surface overexpression of EFG bound significantly more holo-TF in macrophages and significantly improved bacterial intracellular survival ability. Our findings reveal a novel iron acquisition mechanism involving EFG, which suggests novel research avenues into the molecular mechanism of ExPEC resistance to nutritional immunity.

**IMPORTANCE** Extraintestinal pathogenic Escherichia coli (ExPEC) is an important pathogen causing systemic infections in humans and animals. The competition for iron between ExPEC and the host is a determinant for ExPEC to establish a successful infection. Here, we sought to elucidate the role of transferrin (TF) in the interaction between ExPEC and the host. Our results revealed that holo-TF could be utilized by ExPEC to enhance its growth in culture medium and survival in macrophages. Furthermore, the role of elongation factor G (EFG), a novel holo-TF-binding and TF-iron release protein, was confirmed in this study. Our work provides insights into the iron acquisition mechanism of ExPEC, deepens understanding of the interaction between holo-TF and pathogens, and broadens further researches into the molecular mechanism of ExPEC pathogenicity.

## INTRODUCTION

Extraintestinal pathogenic Escherichia coli (ExPEC) is responsible for infections in both humans and farm animals (e.g., urinary tract infections, neonatal meningitis, and sepsis), which impose a substantial burden on both public health and economics (1–3). Compared to commensal E. coli, ExPEC carries additional virulence factors to assist the bacteria in invasion, colonization, survival, and establishment of infection in the host (4). The iron acquisition system is an important virulence factor for ExPEC (5).

Iron is an essential element for nearly all microorganisms and host cells and is required to maintain essential biological functions, including DNA synthesis and repair, transcriptional regulation, and energy metabolism (6). In mammals, free iron is very scarce, as most iron is bound to storage proteins (e.g., hemoglobin, transferrin [TF], and lactoferrin), thus curtailing the availability of iron to pathogens (7). This phenomenon of host iron sequestration is termed nutritional immunity, which represents a facet of innate immunity (8). Thus, to survive and cause disease in a host, bacteria must overcome the challenges of iron sequestration.

TF is the principal iron storage protein found in plasma. In addition, TF is found on mucosal surfaces, as well as in the cerebrospinal fluid (9, 10). Moreover, TF plays an invaluable role in maintaining iron homeostasis in a host. On one hand, free iron is scavenged by TF, which maintains a low-iron antibacterial environment. On the other hand, the iron-carrying TF transports Fe^3+^ to cells through endocytosis processes mediated by TF receptor 1 (6, 11). Pathogens have evolved several strategies to take iron from TF during infection. In addition to secreting iron-chelating siderophores to directly plunder the iron from TF, some bacteria also express TF-binding proteins on their surface to recruit TF (12–15). TbpA and TbpB represent typical TF-binding proteins of Neisseria meningitidis and Neisseria gonorrhoeae. TbpA is a TonB-dependent receptor (TBDR) critical for iron acquisition from TF, whereas TbpB is a surface-exposed lipoprotein that enhances the efficiency of iron uptake (16, 17). In addition, TbpA binds iron-free apo-TF and iron-saturated holo-TF, whereas TbpB discriminates between the two forms and associates specifically with holo-TF (18). Both TbpA and TbpB liberate Fe^3+^ from TF on the surface and transport it into the cytoplasm through TonB and its downstream proteins (10). In Mycobacterium tuberculosis, the surface protein, GAPDH, has been demonstrated to function like a TF-binding protein. GAPDH has been shown to specifically recruit holo-TF rather than apo-TF to the bacterial surface and internalize holo-TF into the cytoplasm in a GAPDH-dependent manner (19). In enteropathogenic E. coli, outer membrane protein A (OmpA) and OmpC are considered to be TF-binding proteins; however, it is unknown whether E. coli can utilize the iron in TF (20).

ExPEC possesses the ability to survive in host serum, cerebrospinal fluid, and macrophages (21, 22). To establish a successful infection, ExPEC must acquire sufficient nutrients, including iron from the host. However, the mechanism by which ExPEC obtains iron has not been well established. We found that ExPEC can directly recruit holo-TF to the surface of the bacteria and that the elongation factor G (EFG), a membrane protein of ExPEC, is a potential holo-TF-binding protein. Subsequently, this study focuses on the utilization of holo-TF by ExPEC and the role of EFG in the interaction between ExPEC and TF, with the aim of revealing a novel mechanism of ExPEC-mediated iron predation in the host.

## RESULTS

### Holo-TF promotes ExPEC RS218 growth in culture medium and survival in macrophages.

**FIG 1:**
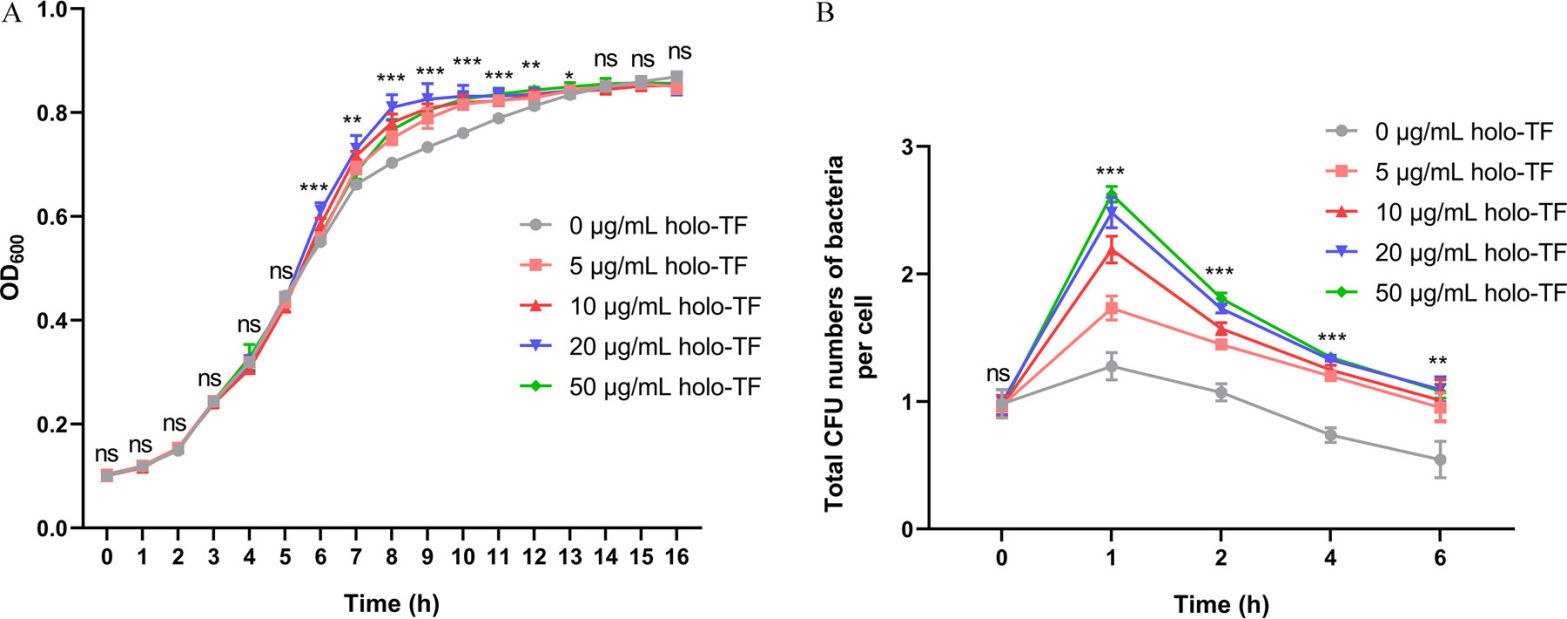
Holo-TF promotes the growth and intracellular survival of ExPEC RS218. (A) The ExPEC strain RS218 was cultured in M9 medium with different concentrations of holo-TF or without holo-TF. The OD_600_ representing the bacterial content was measured each hour. (B) The ExPEC strain RS218 was incubated with THP-1 cells for 1 h, and then the extracellular bacteria were removed. After the cells were incubated with different concentrations of holo-TF or without holo-TF for 0, 1, 2, 4, and 6 h, the numbers of live cells and intracellular bacteria were counted. Total CFU numbers of bacteria per THP-1 cell were calculated as the ratio of the number of bacteria to the number of THP-1 cells. Data are expressed as the mean ± standard deviation. Statistical analyses were assessed using one-way ANOVA. *, *P *<* *0.05; **, *P *<* *0.01; ***, *P *<* *0.001; ns, no significance.

ExPEC strain RS218 is a clinical neonatal meningitis isolate that can cause bacteremia and meningitis (23). To determine the effects of holo-TF on ExPEC, the kinetic curve of RS218 in iron-free M9 minimal culture medium supplemented with holo-TF and the total number of bacteria in per holo-TF-supplemented macrophage were evaluated. As shown in Fig. 1A, the addition of holo-TF significantly promoted bacterial growth at 6 to 13 h (*P *<* *0.05). Dose-dependent effects were observed among different holo-TF concentrations at 6 to 8 h (Fig. S1; *P *<* *0.05). In macrophages, significantly greater numbers of bacteria per cell were isolated in holo-TF-supplemented THP-1 cells (*P *<* *0.05) (Fig. 1B). Dose-dependent effects were observed among different holo-TF concentrations at 1 to 2 h (*P *<* *0.05) (Fig. S2). These results indicated that holo-TF promoted ExPEC RS218 growth in culture medium and survival in macrophages.

### Holo-TF binds ExPEC RS218.

**FIG 2:**
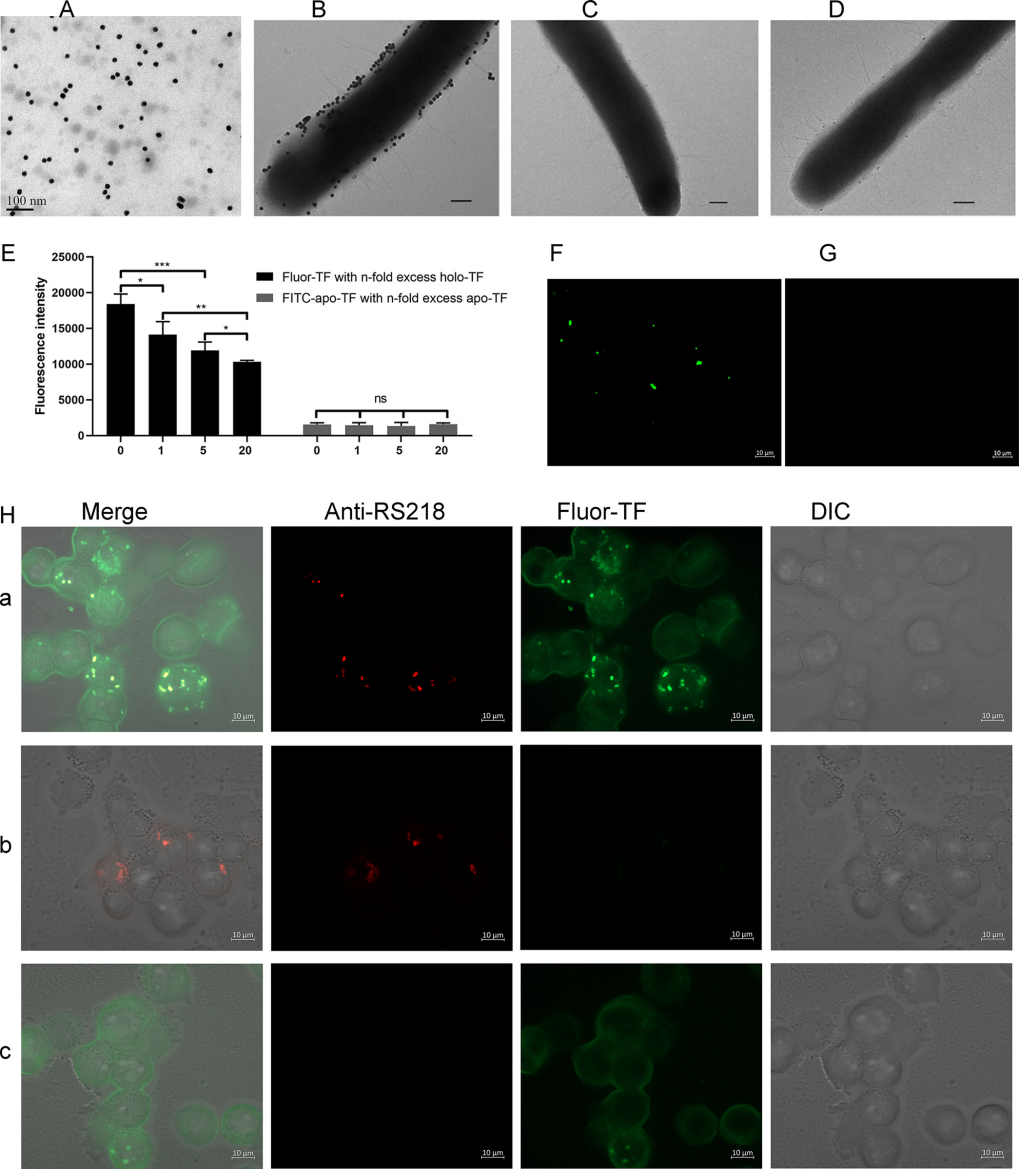
ExPEC RS218 binds to holo-TF in a liquid environment and in macrophages. (A) The preparation of colloidal gold-conjugated holo-TF. ExPEC RS218 was incubated with colloidal gold-conjugated holo-TF (B), colloidal gold-conjugated apo-TF (C), or colloidal gold-conjugated CA (D) at 4°C. The binding of RS218 and colloidal gold-labeled proteins was observed under TEM. Scale bar: 100 nm. (E) Unlabeled holo-TF competed with fluor-TF for binding to ExPEC RS218. RS218 incubated with fluor-TF alone or fluor-TF with different dosages of nonlabeled holo-TF. The fluorescence intensity of RS218-bound fluor-TF was measured at an excitation of 485 nm and an emission of 535 nm. Similarly, RS218 was detected with FITC-apo-TF and apo-TF. *n* = 0, 1, 5, or 20. After incubation with fluor-TF or FITC-apo-TF, the fluorescence signal of fluor-TF (F) and FITC-apo-TF (G) on the surface of the RS218 was detected with a fluorescence microscope. (H) The representative figure for the colocation of fluor-TF and the ExPEC strain in THP-1. a, THP-1 treated with RS218 was incubated with fluor-TF. b, THP-1 cells were incubated with RS218. c, THP-1 cells incubated with fluor-TF. Data are expressed as the mean ± standard error. Statistical analyses for all pairwise comparisons were determined using unpaired *t* test. *, *P *<* *0.05; **, *P *<* *0.01; ***, *P *<* *0.001; ns, no significance.

Holo-TF has been reported to be recruited to the bacterial surface (16). To explore whether holo-TF might bind to ExPEC, the transmission electron microscopy (TEM) assays and fluorescence assays were performed. As shown in Fig. 2A, the colloidal gold-labeled holo-TF exhibited a uniform spherical shape under TEM, with a diameter of approximately 20 nm. It was observed that RS218 was surrounded by holo-TF-conjugated colloidal gold particles (Fig. 2B) instead of apo-TF (Fig. 2C) or carbonic anhydrase (CA; Fig. 2D)-conjugated colloidal gold particles, indicating that holo-TF bound RS218. The specific binding was further confirmed by detecting the competition effect of unlabeled holo-TF on the binding of fluorescein conjugated holo-TF (fluor-TF) to RS218 (*P *<* *0.05) (Fig. 2E), which indicated that the binding of fluor-TF and bacteria was derived from the binding of holo-TF and bacteria, not the binding of fluorescein and bacteria. However, fluorescein isothiocyanate (FITC)-conjugated apo-TF (FITC-apo-TF) does not have the ability to bind bacteria (*P > *0.05). The binding of fluor-TF to the RS218 surface was also detected by fluorescence microscopy (Fig. 2F). In addition, the interaction between intracellular bacteria and holo-TF was determined. As shown in Fig. 2H, the colocalization of RS218 and fluor-TF in THP-1 cells indicated that ExPEC RS218 recruited holo-TF in macrophages.

### ExPEC RS218 acquires iron from holo-TF.

**FIG 3:**
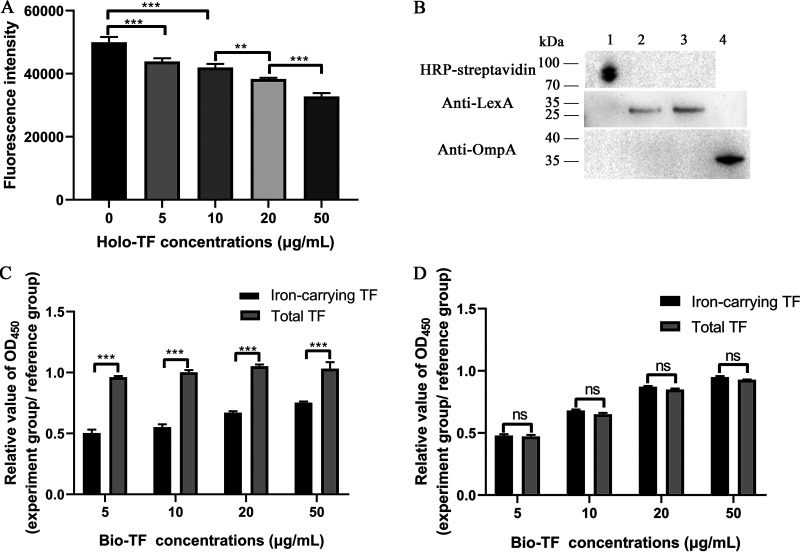
ExPEC RS218 acquires iron in holo-TF. (A) Calcein-AM fluorescence quenching assays. RS218 was stained with calcein. The quenching of fluorescence of calcein indicating iron uptake was measured at 37°C in the absence or presence of different concentrations of holo-TF. Data from 10^7^ cells were acquired at an excitation of 490 nm and an emission of 538 nm. (B) Blotting assays for the detection of TF in bacterial cytoplasm. RS218 and bio-TF were incubated at 37°C (lane 2) or 4°C (lane 3). The bio-TF in cytosolic fractions was collected and detected using HRP-streptavidin. The LexA and OmpA signals in cytosolic fractions of ExPEC were also detected by Western blotting using anti-LexA and OmpA antibodies. Bio-TF was used as a positive control for the detection of holo-TF (lane 1). The outer membrane fractions of ExPEC were used as a positive control for the detection of OmpA (lane 4). (C and D) Holo-TF conversion detection assays. RS218 was incubated with different concentrations of bio-TF at 37°C (C) or 4°C (D). The OD_450_ values of desthiobiotin (representing total TF) and iron-carrying TF signals in the supernatant were measured using HRP-streptavidin and anti-TF antibodies, respectively. The same concentration of bio-TF was used as the reference. Relative values of OD_450_ of total TF signal were calculated as the ratio of the total TF signals of the experimental group to that of the reference. Similarly, the relative OD_450_ value of the iron-carrying TF signals was also calculated. Data are expressed as the mean ± standard error. Statistical analyses for all pairwise comparisons were determined using unpaired *t* test. **, *P *<* *0.01; ***, *P *<* *0.001; ns, no significance.

Bacteria have been reported to take up the iron in holo-TF (24). To detect whether holo-TF-associated iron might be acquired by ExPEC RS218, the calcein-quenching assays were performed. When calcein-AM is taken up by living cells, it can be cleaved by intracellular esterase, which releases calcein into the cytoplasm. The fluorescence of calcein can be quenched by some metal ions (e.g., Fe^3+^) but not by Ca^2+^ or Mg^2+^. As shown in Fig. 3A, the holo-TF supplement significantly reduced the fluorescence intensity of calcein (*P *<* *0.05), which indicated that iron had been acquired. With increasing holo-TF concentrations, the iron acquisition of bacteria significantly increased (*P *<* *0.01).

M. tuberculosis has been reported to recruit holo-TF to the cytoplasm to acquire iron (19). To explore whether RS218 might obtain iron by acquiring holo-TF, EZ-Link sulfo-NHS-LC-desthiobiotin-labeled holo-TF (bio-TF) signals in the cytoplasm of RS218 were detected. No TF band was detected in the cytoplasm, indicating that ExPEC RS218 did not take up holo-TF (Fig. 3B). Western blotting of the extracted bacterial cytoplasmic components showed that LexA, a cytoplasmic protein of E. coli, was positive and OmpA protein was negative, indicating that the extracted bacterial components were indeed cytoplasmic components and did not contain membrane protein contamination (Fig. 3B). This result combined with the above results suggested that ExPEC RS218 recruited holo-TF to acquire iron instead of transporting holo-TF to the cytoplasm.

Iron uptake was also determined by detecting the production of apo-TF. Only iron-carrying TF (holo-TF and partially saturated TF) instead of apo-TF was detected by enzyme-linked immunosorbent assay (ELISA) using anti-TF antibodies (Abcam, USA). Therefore, the iron uptake event could be characterized through comparison of the difference of relative desthiobiotin signals (representing total TF) and relative iron-carrying TF signals after RS218 was incubated with bio-TF. As shown in Fig. 3C, the iron-carrying TF signals were significantly lower than the total TF signals at 37°C, indicating that ExPEC RS218 converted holo-TF to apo-TF (*P *<* *0.05). However, there was no significant difference when bacteria were incubated at 4°C, indicating that ExPEC RS218 cannot convert holo-TF at 4°C (*P *<* *0.05) (Fig. 3D). Furthermore, when RS218 was incubated with bio-TF at 37°C, the total TF signal levels were similar to that in the reference (*P > *0.05), whereas the total TF signals significantly decreased following incubation at 4°C (*P *<* *0.05). This finding indicated that the conversion of holo-TF to apo-TF by RS218 should be performed at 37°C instead of 4°C. Bacterial binding to holo-TF occurred at 4°C. Moreover, when RS218 was incubated with bio-TF at 37°C, the total TF signal levels were similar to that in the reference (*P > *0.05), whereas iron-carrying TF signals levels were significantly lower than the reference signals (*P *<* *0.05), indicating that converted apo-TF detached from the bacteria and was released into the supernatant (Fig. 3C).

The above results indicated that ExPEC RS218 bound to holo-TF and acquired the TF-associated iron. This process did not involve the transport of holo-TF to the cytoplasm. After the iron of holo-TF was plundered by RS218, apo-TF detached from the surfaces of bacteria.

### Holo-TF binds Dm1 and Dm2 of EFG.

The desthiobiotin pulldown assay from ExPEC RS218 total membrane fractions to determine the identity of holo-TF-binding protein(s) indicated the presence of proteins (results and materials and methods in the supplemental material, see Fig. S3). The subsequent mass spectrometry analysis indicated that EFG is one of the proteins with the largest number of matched peptides (Table S1), so it was selected for further research.

**FIG 4:**
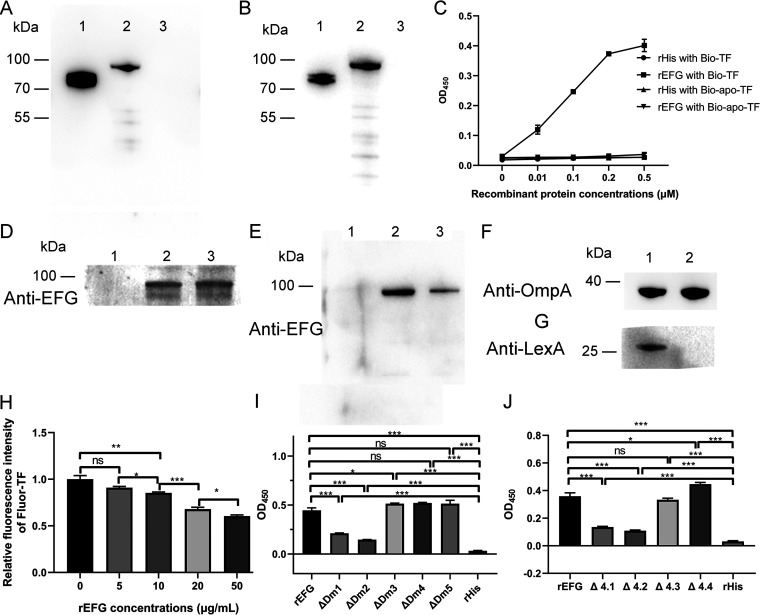
EFG utilizes its N-terminal domains to interact with holo-TF. (A) Far-Western blotting of EFG with holo-TF. Holo-TF (lane 1), rEFG (lane 2), and rHis (negative control, lane 3) were loaded on the PVDF membrane and then overlaid with holo-TF. The interactions were detected using anti-TF antibodies. (B) Holo-TF (lane 1), rEFG (lane 2), and rHis (negative control, lane 3) were loaded on the PVDF membrane and then overlaid with rEFG. The interactions were detected using anti-EFG antibodies. (C) Different concentrations of rEFG or rHis coated on an ELISA plate were incubated with bio-TF or bio-apo-TF. Interactions were detected using HRP-streptavidin. (D) bio-CA and bio-TF were incubated with streptavidin agarose resins and then incubated with rEFG. The resulting complexes containing bio-CA (lane 1) and bio-TF (lane 2) were subjected to Western blotting using anti-EFG antibodies. rEFG was used as a positive control (lane 3). (E) Bio-CA and bio-TF were incubated with streptavidin agarose resins and then incubated with total membrane proteins of ExPEC RS218. The resulting complexes containing bio-CA (lane 1) and bio-TF (lane 2) were subjected to Western blotting using anti-EFG antibodies. Total membrane proteins of RS218 were used as a positive control. (F and G) The total membrane proteins of RS218 (lane 2) for desthiobiotin pulldown assays were detected by Western blotting using anti-OmpA antibodies (F) and anti-LexA antibodies (G). Total proteins of RS218 (lane 1) were used as a positive control. (H) Holo-TF-binding inhibition assays by rEFG. Fluor-TF was preincubated with different concentrations of rEFG and then interacted with RS218. As a reference, fluor-TF was incubated directly with RS218. The fluorescence intensity of the fluor-TF bound on RS218 was detected at an excitation of 485 nm and an emission of 535 nm. Data were determined as the ratio of the fluorescence intensity of the rEFG preincubation group compared with that of the reference group. (I and J) ΔDm1 to ΔDm5, Δ4.1 to Δ4.4, rEFG, and rHis coated on an ELISA plate were incubated with bio-TF. Their interactions were detected using HRP-streptavidin. Data in panel C are expressed as the mean ± standard deviation. Data in panels H, I, and J are expressed as the mean ± standard error. Statistical analyses for all pairwise comparisons were determined using unpaired *t* test. *, *P *<* *0.05; **, *P *<* *0.01; ***, *P *<* *0.001; ns, no significance.

To verify the holo-TF-binding ability of EFG, recombinant EFG (rEFG) was expressed (Fig. S4) and the interaction between rEFG and holo-TF was detected by far-Western blotting, ELISA plate binding assays, desthiobiotin pulldown assays, and recombinant protein inhibition assays. The results of far-Western blotting detected that the holo-TF signal was associated with the rEFG on the polyvinylidene difluoride (PVDF) membrane and the rEFG signal was associated with the holo-TF on the PVDF membrane (Fig. 4A and B). No recombinant His (rHis, obtained from E. coli BL21 carrying the pET-32a plasmid) bands interacting with holo-TF or rEFG were detected, indicating that the interaction between EFG and holo-TF was specific. The results of the ELISA plate binding assays indicated that rEFG bound to bio-TF in a concentration-dependent manner (Fig. 4C). In addition, there was no interaction between rEFG and desthiobiotin-conjugated apo-TF (bio-apo-TF). These results indicated that EFG binds only holo-TF, not apo-TF and desthiobiotin. The results of desthiobiotin pulldown assays demonstrated that bio-TF captured the rEFG as well as EFG in the total membrane proteins extracted from ExPEC strain RS218 (Fig. 4D and E, lane 2). Meanwhile, the total membrane proteins extracted from RS218 were verified and determined to be free of cytoplasmic protein contamination (Fig. 4F and G). The results of recombinant protein inhibition assays showed that preincubation of rEFG and fluor-TF significantly reduced the binding intensity of fluor-TF on the surface of RS218 (*P *<* *0.05) (Fig. 4H).

The domains of EFG were predicted by Pfam (http://pfam.xfam.org/), and the results indicated that EFG contains five domains: the GTP_EFTU (amino acids 8 to 288), GTP_EFTU_D2 (Dm2, amino acids 331 to 398), EFG_III (Dm3, amino acids 411 to 485), EFG_IV (Dm4, amino acids 486 to 609), and EFG_C (amino acids 611 to 689). In this study, amino acids 1 to 288 were regarded as Dm1 and amino acids 611 to 704 were regarded as Dm5. The schematic diagram of domains is showed in Fig. S5. To identify TF-binding domains in EFG, recombinant proteins with domain deletions (ΔDm1, ΔDm2, ΔDm3, ΔDm4, and ΔDm5) were constructed (Fig. S5B). The results of ELISA binding assays in Fig. 4I showed that the deletion of Dm1 and Dm2 significantly reduced the holo-TF-binding ability of EFG (*P *<* *0.01), which indicated that Dm1 and Dm2 of EFG were key regions of EFG binding to holo-TF.

Next, to identify TF-binding regions in Dm1, the Dm1 were divided into four regions (4.1, 4.2, 4.3, and 4.4; Fig. S5A), and recombinant proteins deficient in each region were constructed (Fig. S5B). The results in Fig. 4J indicated that regions 4.1 and 4.2 were key regions for the Dm1 binding to holo-TF.

The above results indicated that the EFG interacted with holo-TF via its N-terminal domains (Dm1 [especially amino acids 1 to 142] and Dm2).

### The binding of holo-TF and EFG leads to iron release.

**FIG 5:**
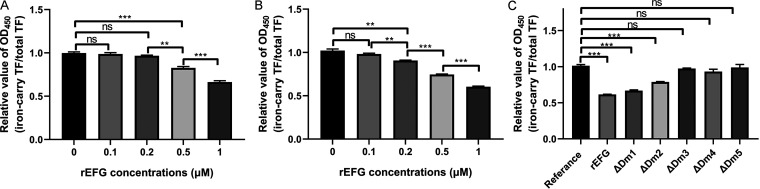
EFG utilizes its C-terminal domains to release iron from holo-TF. Bio-TF was incubated with different concentrations of rEFG at 4°C (A) or 37°C (B). Then, protein mixtures and the same concentration of bio-TF (reference group) were coated on an ELISA plate. The total TF signals and iron-carrying TF signals were detected using HRP-streptavidin and anti-TF antibodies, respectively. The relative value of OD_450_ was calculated as the ratio of OD_450_ value of iron-carrying TF signals to that of total TF signals. (C) ΔDm1 to ΔDm5 and rEFG were incubated with bio-TF at 37°C. The total TF signals and iron-carrying TF signals were detected and relative values were measured as described above. Data are expressed as the mean ± standard error. Statistical analyses for all pairwise comparisons were determined using unpaired *t* test. **, *P *<* *0.01; ***, *P *<* *0.001; ns, no significance.

The classical TF-binding proteins TbpA and TbpB have been identified to release iron in holo-TF (25, 26). To explore the ability of EFG to release TF-associated iron, iron-carrying TF and desthiobiotin signals after the rEFG and bio-TF interaction were detected. If iron is released after the holo-TF interaction with EFG, holo-TF converts to partially iron-saturated TF and even apo-TF, resulting in a decrease in iron-carrying TF signals. The intensity of the desthiobiotin signal representing total TF is not affected by the form of TF. The iron release event can be detected by calculation of the ratio of the signal of the iron-carrying TF to the signal of total TF. As shown in Fig. 5A, when the rEFG and bio-TF were incubated at 4°C for 2 h, the relative values of optical density at 450 nm (OD_450_; iron-carrying TF/total TF) were significantly lower in the 0.5 μM rEFG and 1 μM rEFG groups than in the reference group (*P *<* *0.05). When incubated at 37°C, the relative values of OD_450_ were significantly lower in the 0.2, 0.5, and 1 μM rEFG groups than in the reference group (*P *<* *0.05) (Fig. 5B), indicating that rEFG released iron from holo-TF in a dose-dependent manner.

The key domains of EFG associated with iron release were also explored. As shown in Fig. 5C, compared with those in the reference group, the relative values of OD_450_ (iron-carrying TF/total TF) of rEFG, ΔDm1, and ΔDm2 were significantly lower (*P *<* *0.01), which indicated the production of apo-TF. The relative values of OD_450_ of ΔDm3, ΔDm4, and ΔDm5 were not significantly different from those of the reference group (*P > *0.05), indicating that the deletion of Dm3, Dm4, and Dm5 of EFG resulted in the failure of apo-TF generation. These results suggested that the C-terminal domains of EFG, including Dm3, Dm4, and Dm5, were involved in the process of iron release after binding with holo-TF.

### EFG locates on the surface of ExPEC RS218.

**FIG 6:**
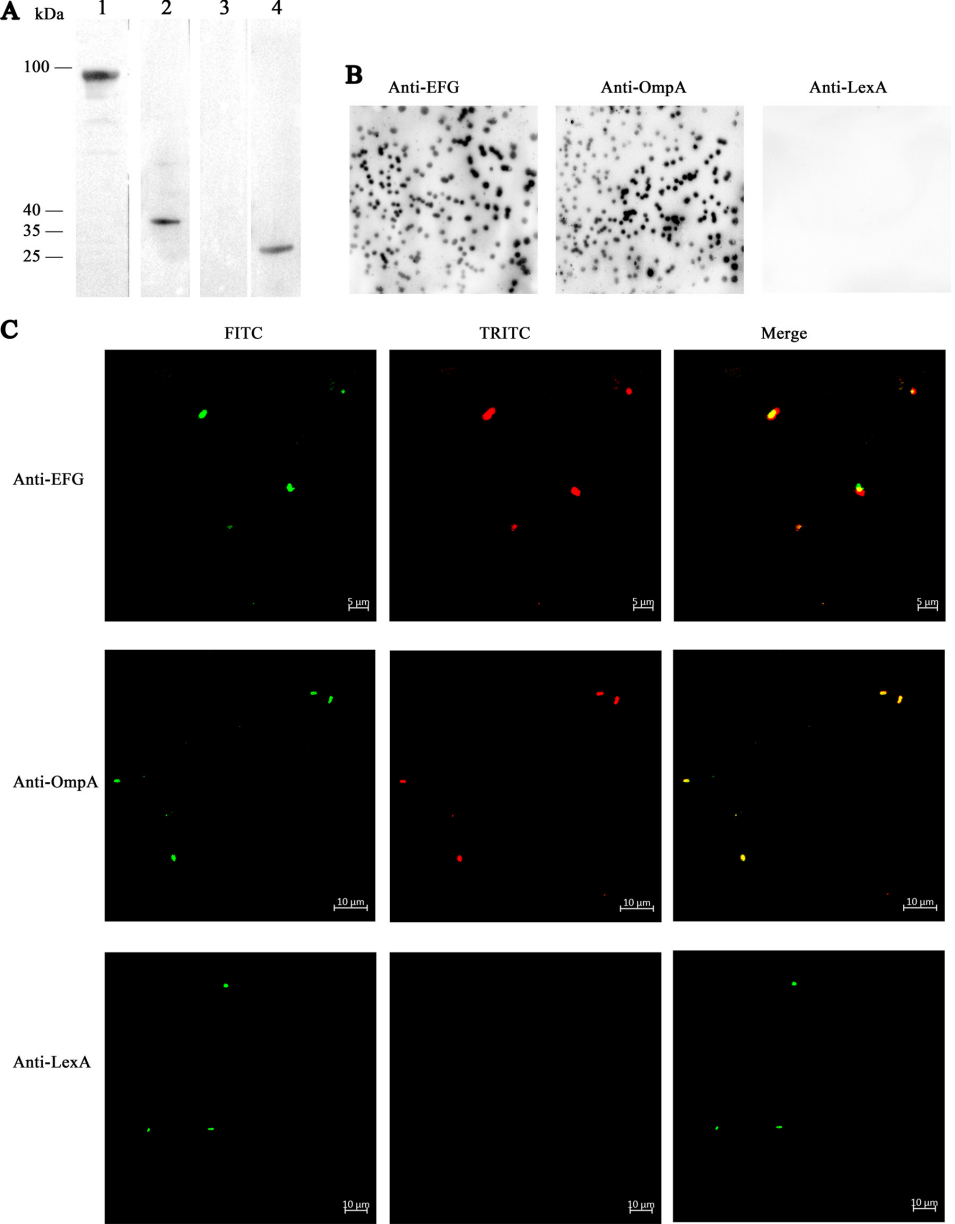
EFG locates on the surface of ExPEC RS218. (A) The RS218 outer membrane proteins were isolated and detected by Western blotting using anti-EFG (lane 1), anti-OmpA (lane 2, positive control for outer membrane proteins), or anti-LexA (lane 3, positive control for cytoplasmic proteins) antibodies. Cytoplasmic proteins were also detected using anti-LexA antibodies (lane 4). (B) Colony blotting assay of RS218. The nitrocellulose membrane was in contact with bacterial colonies. Proteins stained on the nitrocellulose membrane were detected using anti-EFG, anti-OmpA, and anti-LexA antibodies. (C) The RS218 strain was treated with a mixture of mouse anti-RS218 antiserum and anti-EFG, anti-OmpA, or anti-LexA antibodies. Detection was conducted with FITC-conjugated anti-mouse IgG and TRITC-conjugated anti-rabbit IgG incubation.

The reported bacterial TF-binding proteins (e.g., TbpA, TbpB, and GAPDH) are surface proteins. To investigate whether EFG might be located on the surface of RS218, Western blotting, colony blotting, and immunofluorescence assays were performed. The positive signals of EFG and OmpA on RS218 outer membrane fractions (Fig. 6A) and the colony surface (Fig. 6B) indicated that EFG was a surface protein. A negative LexA signal excluded cytoplasmic protein contamination. The presence of obvious FITC signal corresponding to the detection of RS218 strain and tetramethyl rhodamine isocyanate (TRITC) signals corresponding to the detections of EFG and OmpA, as well as the absence of the TRITC signal corresponding to the detection of LexA in Fig. 6C, also suggested the surface localization of EFG.

### Construction and verification of strains with surface-display of heterologous proteins.

**FIG 7:**
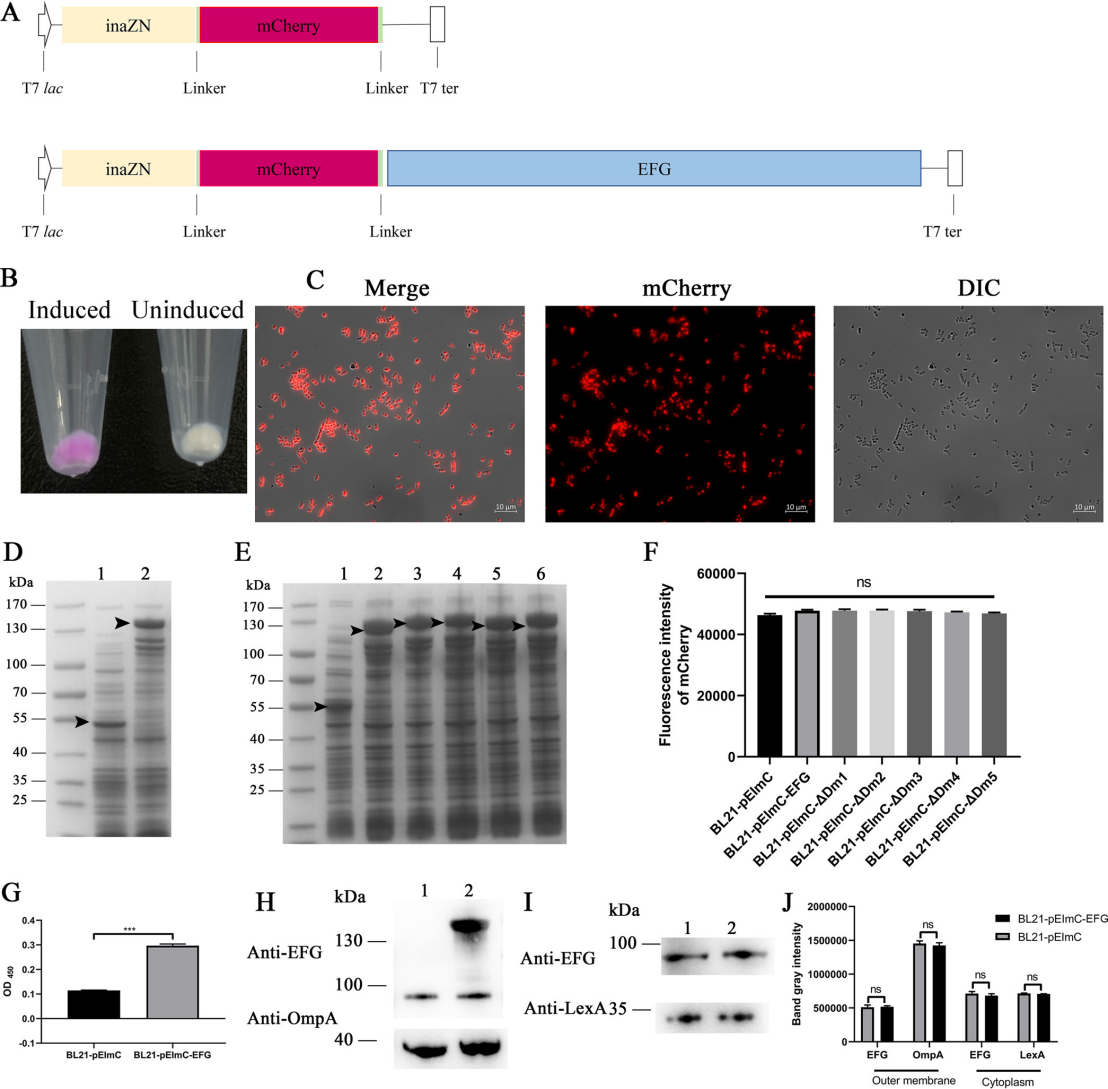
Availability of the surface-displaying strains. (A) Schematic diagram of the pEImC and pEImC::*efg* plasmids. (B) Visual observation of pellets of induced and uninduced BL21-pEImC strains. (C) The induced BL21-pEImC strains were spread on the cover glass and observed through the DIC channel and mCherry channel of a fluorescence microscope. (D) SDS-PAGE analysis of the induced total bacterial proteins of BL21-pEImC (lane 1) and BL21-pEImC-EFG (lane 2). (E) SDS-PAGE analysis of the induced total bacterial proteins of BL21-pEImC (lane 1), BL21-pEImC-ΔDm1 (lane 2), BL21-pEImC-ΔDm2 (lane 3), BL21-pEImC-ΔDm3 (lane 4), BL21-pEImC-ΔDm4 (lane 5), and BL21-pEImC-ΔDm5 (lane 6). The black arrow indicates the induced protein bands. (F) The fluorescence intensity of mCherry of seven BL21 strains containing a series of pEImC plasmids was measured at an excitation of 579 nm and an emission of 624 nm. (G) Whole bacteria of induced BL21-pEImC and BL21-pEImC-EFG were coated on an ELISA plate. The surface expressed EFG was detected by ELISA using anti-EFG antibodies. (H) The outer membrane fractions of the BL21-pEImC strain (lane 1) and BL21-pEImC-EFG strain (lane 2) were detected by Western blotting using anti-EFG and anti-OmpA antibodies. (I) The cytoplasmic fractions of the BL21-pEImC (lane 1) and BL21-pEImC-EFG (lane 2) were detected by Western blotting using anti-EFG and anti-LexA antibodies. (J) Gray intensity analysis of endogenous EFG, OmpA, and LexA bands in panels H and I. Data are expressed as the mean ± standard error. Statistical analyses in panel F were determined using one-way ANOVA and in panels G and J were determined using unpaired *t* test, respectively. ***, *P *<* *0.001; ns, no significance.

The pEImC plasmid for heterologous protein surface-display was constructed according to previous studies with modifications (27–29). Gene maps of pEImC and pEImC::*efg* are presented in Fig. 7A. The N-terminal domain of the ice nucleation protein (InaZN, accession no. PBP57058) was used as the anchoring motif to enable display of heterologous proteins on the surfaces of E. coli. The mCherry protein (GenBank: MK753226.1) was used as an indicator of protein expression on the surfaces. The fusion expression of EFG or its variants with InaZN and mCherry enabled heterologous expression of EFG or its variants on the surfaces of bacteria.

The pEImC and its recombinant plasmids, including pEImC::*efg*, pEImC::ΔDm1, pEImC::ΔDm2, pEImC::ΔDm3, pEImC::ΔDm4, and pEImC::ΔDm5, were transformed into E. coli BL21(DE3). The obtained strains were named BL21-pEImC, BL21-pEImC-EFG, BL21-pEImC-ΔDm1, BL21-pEImC-ΔDm2, BL21-pEImC-ΔDm3, BL21-pEImC-ΔDm4, and BL21-pEImC-ΔDm5, respectively (Table 1).

**TABLE 1:** Bacterial strains and plasmids used in this study

Bacterial strain or plasmid	Notable characteristic(s)^*a*^	Source or reference
Bacteria		
DH5α	Host for plasmid cloning	Purchased from Vazyme
BL21(DE3)	Host for recombinant protein expression	Purchased from Vazyme
RS218	Virulent strain of ExPEC from the cerebrospinal fluid in neonates with meningitis	23
BL21-pEImC	BL21(DE3) strain containing pEImC plasmid	This study
BL21-pEImC-EFG	BL21(DE3) strain containing pEImC::*efg* plasmid	This study
BL21-pEImC-ΔDm1	BL21(DE3) strain containing pEImC::ΔDm1 plasmid	This study
BL21-pEImC-ΔDm2	BL21(DE3) strain containing pEImC::ΔDm2 plasmid	This study
BL21-pEImC-ΔDm3	BL21(DE3) strain containing pEImC::ΔDm3 plasmid	This study
BL21-pEImC-ΔDm4	BL21(DE3) strain containing pEImC::ΔDm4 plasmid	This study
BL21-pEImC-ΔDm5	BL21(DE3) strain containing pEImC::ΔDm5 plasmid	This study
Plasmids		
pET-32a	Prokaryotic recombinant protein expression plasmid, Amp^r^	Purchased from Takara
pET-28a	Prokaryotic recombinant protein expression plasmid, Kan^r^	Purchased from Takara
pET-28a::*efg*	pET-28a containing *efg*, Kan^r^	This study
pET-28a::ΔDm1	pET-28a containing *efg* with Dm1 deletion, Kan^r^	This study
pET-28a::ΔDm2	pET-28a containing *efg* with Dm2 deletion, Kan^r^	This study
pET-28a::ΔDm3	pET-28a containing *efg* with Dm3 deletion, Kan^r^	This study
pET-28a::ΔDm4	pET-28a containing *efg* with Dm4 deletion, Kan^r^	This study
pET-28a::ΔDm5	pET-28a containing *efg* with Dm5 deletion, Kan^r^	This study
pET-28a::ΔD4.1	pET-28a containing *efg* with amino acids 1–72 deletion, Kan^r^	This study
pET-28a::ΔD4.2	pET-28a containing *efg* with amino acids 73–142 deletion, Kan^r^	This study
pET-28a::ΔD4.3	pET-28a containing *efg* with amino acids 143–228 deletion, Kan^r^	This study
pET-28a::ΔD4.4	pET-28a containing *efg* with amino acids 229–288 deletion, Kan^r^	This study
pEImC	Surface-display plasmid, Kan^r^	This study
pEImC::*efg*	pEImC containing *efg*, Kan^r^	This study
pEImC::ΔDm1	pEImC containing *efg* with Dm1 deletion, Kan^r^	This study
pEImC::ΔDm2	pEImC containing *efg* with Dm2 deletion, Kan^r^	This study
pEImC::ΔDm3	pEImC containing *efg* with Dm3 deletion, Kan^r^	This study
pEImC::ΔDm4	pEImC containing *efg* with Dm4 deletion, Kan^r^	This study
pEImC::ΔDm5	pEImC containing *efg* with Dm5 deletion, Kan^r^	This study

a Kan^r^, kanamycin resistance; Amp^r^, ampicillin resistance.

Compared to uninduced BL21-pEImC, the IPTG (isopropyl-β-d-thiogalactopyranoside)-induced bacterial pellet turned bright fuchsia (Fig. 7B). Fluorescence microscopy observed that the induced BL21-pEImC had strong fluorescence signals (Fig. 7C). SDS-PAGE of whole bacterial proteins of induced BL21-pEImC, BL21-pEImC-EFG, and BL21-pEImC-ΔDm1 to BL21-pEImC-ΔDm5 is shown in Fig. 7D and E. The overexpression bands indicated the successful expression of fusion proteins. The detection of the fluorescence intensity of mCherry showed that the level of mCherry expression of these seven BL21 strains containing a series of pEImC plasmids (BL21-pEImC, BL21-pEImC-EFG, and BL21-pEImC-ΔDm1 to BL21-pEImC-ΔDm5) showed no significant differences (*P *<* *0.05) (Fig. 7F). Whole bacteria ELISA indicated that the level of EFG surface expression in BL21-pEImC-EFG was significantly higher than that in BL21-pEImC (*P *<* *0.01) (Fig. 7G). Outer membrane proteins and cytoplasmic proteins in BL21-pEImC and BL21-pEImC-EFG were detected. As shown in Fig. 7H, in the outer membrane fractions, the fusion expressed EFG signal (approximately 130 kDa) was detected in BL21-pEImC-EFG, whereas the endogenous EFG band (approximately 100 kDa) was detected in both BL21-pEImC-EFG and BL21-pEImC. In the cytoplasmic fractions, bands of endogenous EFG (approximately 100 kDa) and LexA (approximately 25 kDa) were detected in both BL21-pEImC and BL21-pEImC-EFG (Fig. 7I). Gray intensity analyses indicated that the surface expression levels of the endogenous EFG and other proteins of the two strains were not significantly different (*P > *0.05) (Fig. 7J). These results indicated that the fusion expression of EFG on the bacterial surfaces did not affect the expression of endogenous EFG on the surfaces and in the cytoplasm, as well as that of other endogenous proteins, and also excluded the influence of endogenous EFG on subsequent experiments.

### The binding of EFG and holo-TF facilitates iron absorption in E. coli.

**FIG 8:**
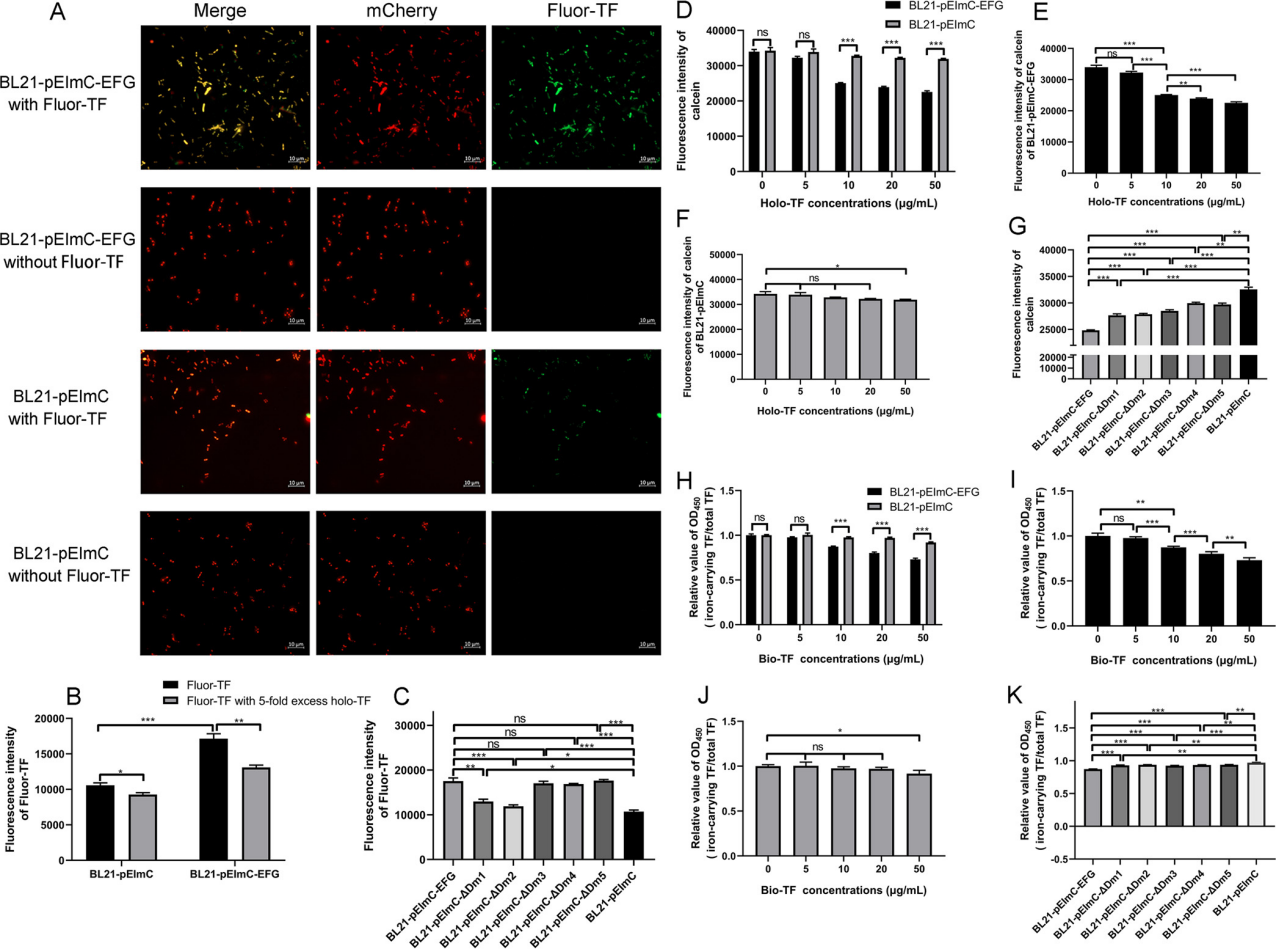
The overexpression of EFG on the E. coli surface promotes the binding of holo-TF and uptake of holo-TF-related iron by bacteria. (A) After BL21-pEImC-EFG and BL21-pEImC were incubated with fluor-TF, the fluorescence of the bound fluor-TF was observed under a fluorescence microscope. BL21-pEImC-EFG and BL21-pEImC incubated without fluor-TF were used as negative controls. (B) Unlabeled holo-TF competed with fluor-TF for binding to BL21-pEImC and BL21-pEImC-EFG. BL21-pEImC and BL21-pEImC-EFG were incubated with fluor-TF alone or with a mixture of fluor-TF and holo-TF. The fluorescence intensity of fluor-TF bound to the bacterial surface from 10^7^ bacterial cells was detected at an excitation of 485 nm and an emission of 535 nm. (C) Seven BL21 strains containing a series of pEImC plasmids were incubated with 20 μg/mL fluor-TF. The fluorescence intensity of fluor-TF bound to the surface of 10^7^ bacterial cells was detected. (D) The quenching of the fluorescence of calcein, which stained in the BL21-pEImC-EFG and BL21-pEImC strains, was measured at 37°C in the presence of different concentrations of holo-TF. The fluorescence intensity of calcein from 10^7^ bacterial cells was detected at an excitation of 490 nm and an emission of 538 nm. (E and F) Separate analysis of the results described in the legend of panel D regarding the fluorescence intensity of calcein for the BL21-pEImC-EFG and the BL21-pEImC strains. (G) Seven BL21 strains containing a series of pEImC plasmids were stained with calcein and incubated with holo-TF at 37°C. The fluorescence intensity of calcein from 10^7^ bacterial cells of different strains was detected. (H) The BL21-pEImC-EFG and BL21-pEImC strains were incubated with different concentrations of bio-TF. Proteins in the supernatant were coated on an ELISA plate. Total TF signals and iron-carrying TF signals were detected using HRP-streptavidin and anti-TF antibodies, respectively. The relative value was calculated as the ratio of the iron-carrying TF signals to the total TF signals. (I and J) Separate analysis of the results described in the legend of panel H regarding the relative value of the iron-carrying TF signals to the total TF signals for the BL21-pEImC-EFG and the BL21-pEImC strains. (K) Seven BL21 strains containing a series of pEImC plasmids were incubated with 10 μg/mL bio-TF. The relative values of iron-carrying TF signals to the total TF signals of each strain were calculated. Data are expressed as the mean ± standard error. Statistical differences were determined using unpaired *t* test. *, *P *<* *0.05; **, *P *<* *0.01; ***, *P *<* *0.001; ns, no significance.

Our study revealed that the binding of rEFG and holo-TF facilitated iron release in phosphate-buffered saline (PBS). To detect the interaction between EFG and holo-TF at bacterial level, the binding of fluor-TF to BL21 strains containing a series of pEImC plasmids was detected. As shown in Fig. 8A, obvious fluor-TF signals were observed in BL21-pEImC-EFG, whereas there were weak signals in BL21-pEImC strain. No fluor-TF signals were detected in the bacteria that were not incubated with fluor-TF, excluding the fluorescence crossover between mCherry and fluor-TF. In addition, the fluorescence intensity of the fluor-TF on the surfaces of BL21-pEImC-EFG was significantly higher than that of the BL21-pEImC (*P *<* *0.001) (Fig. 8B). An excess of unlabeled holo-TF significantly reduced the fluorescence intensity of BL21-pEImC-EFG to 76.38%, whereas fluorescence of BL21-pEImC significantly reduced by only 12.24% (*P *<* *0.05) (Fig. 8B), thus excluding the binding of fluorescein and bacteria. The fluor-TF-binding ability of BL21-pEImC-ΔDm1 to BL21-pEImC-ΔDm5 is showed in Fig. 8C. Compared with that of BL21-pEImC, the fluorescence intensity of fluor-TF bound to BL21-pEImC-EFG and BL21-pEImC-ΔDm1 to BL21-pEImC-ΔDm5 was significantly greater (*P *<* *0.01). However, the fluorescence intensity of fluor-TF bound to BL21-pEImC-ΔDm1 and BL21-pEImC-ΔDm2 was significantly lower than that of BL21-pEImC-EFG (*P *<* *0.01), which indicated that Dm1 and Dm2, not the Dm3 to Dm5 of EFG, were involved in holo-TF binding.

To explore the role of EFG in iron uptake at the bacterial level, the calcein-quenching assays were performed on BL21 strains containing a series of pEImC plasmids. The quenching of the fluorescence intensity of calcein indicated the bacterial uptake of the TF-related iron. The results in Fig. 8D to F indicated that the iron uptake ability of BL21-pEImC-EFG was significantly stronger than that of BL21-pEImC strain (*P *<* *0.05). BL21-pEImC-EFG acquired TF-related iron in a concentration-dependent manner (*P* < 0.01) (Fig. 8E). The fluorescence intensity of calcein of BL21-pEImC-ΔDm1 to BL21-pEImC-ΔDm5 was significantly higher than that of BL21-pEImC-EFG, indicating that their iron uptake ability was significantly lower than that of BL21-pEImC-EFG (*P *< 0.001) (Fig. 8G). In addition, the production of apo-TF after bacteria were incubated with bio-TF was detected. As shown in Fig. 8H to J, the relative value (iron-carrying TF/total TF) of the BL21-pEImC-EFG was significantly lower than that of the BL21-pEImC strain (*P *<* *0.001), indicating that overexpressed EFG significantly promoted the conversion of holo-TF to apo-TF. The BL21-pEImC-EFG converted holo-TF to apo-TF in a bio-TF concentration-dependent manner (*P* < 0.01) (Fig. 8I). For BL21-pEImC-ΔDm1 to BL21-pEImC-ΔDm5, the ability to transform holo-TF was significantly weaker than that of the BL21-pEImC-EFG strain (*P *<* *0.001) (Fig. 8K).

These results indicated that the overexpression of EFG on the bacterial surfaces was positively correlated with holo-TF-binding and iron absorption ability. The N-terminal domain of EFG played an important role in the binding of E. coli to holo-TF. The deletion of iron-releasing domains and the holo-TF-binding domains in EFG suppressed the iron uptake capacity on the whole bacterium level.

### The binding of EFG and holo-TF promotes the survival of E. coli in macrophages.

**FIG 9:**
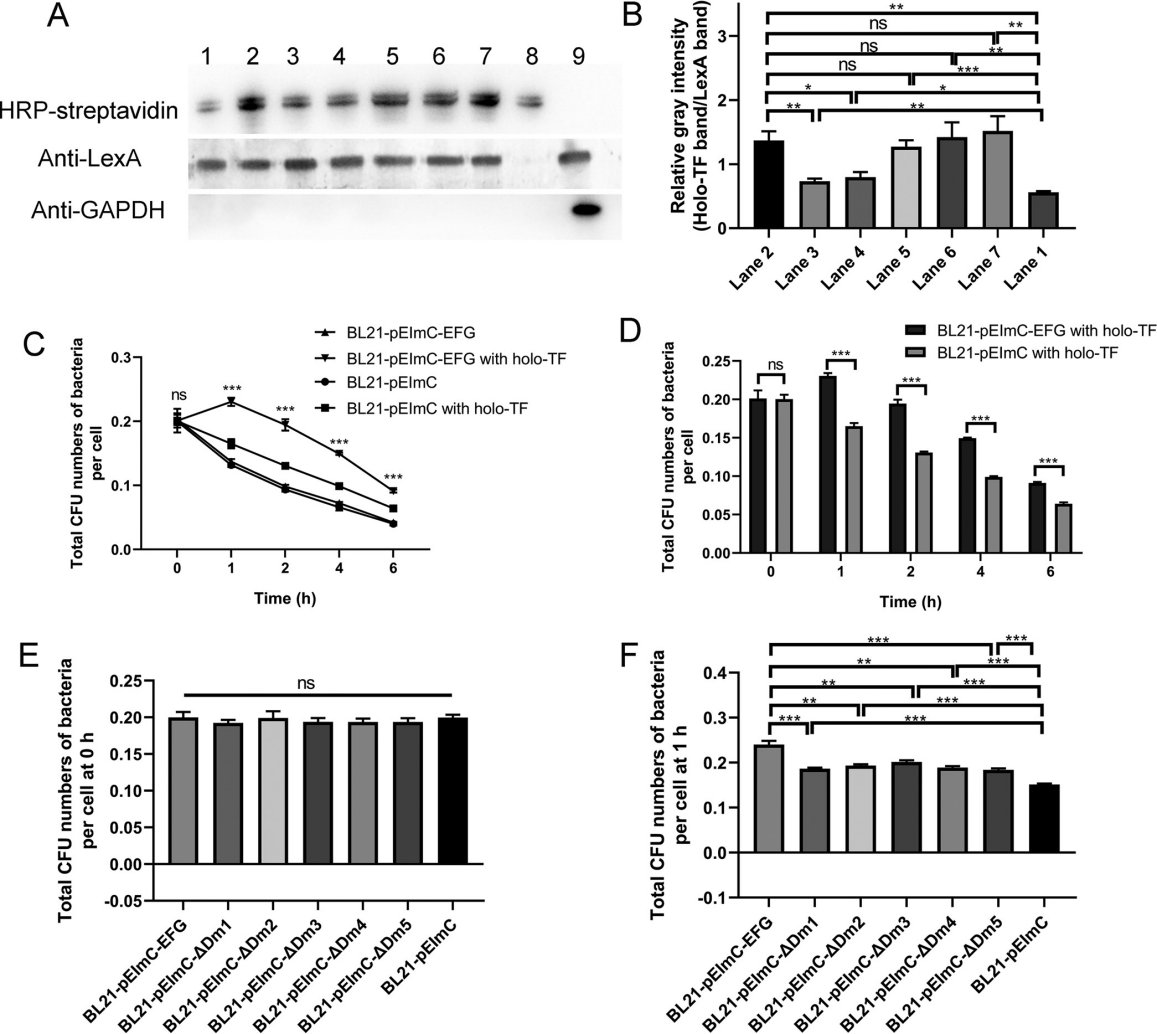
The overexpression of EFG on the E. coli surface promotes the binding of bacteria to intracellular holo-TF and survival ability in macrophages. (A) Isolated intracellular BL21 strains containing a series of pEImC plasmids combined with bio-TF in THP-1 were detected using HRP-streptavidin. The isolated bacteria were determined by detection of LexA using anti-LexA antibodies. The bacteria were isolated from the THP-1 cells whether or not carryover of cellular holo-TF was determined by detection of GAPDH using anti-GAPDH antibodies. Lane 1, isolated BL21-pEImC; lane 2, isolated BL21-pEImC-EFG; lane 3, isolated BL21-pEImC-ΔDm1; lane 4, isolated BL21-pEImC-ΔDm2; lane 5, isolated BL21-pEImC-ΔDm3; lane 6, isolated BL21-pEImC-ΔDm4; lane 7, isolated BL21-pEImC-ΔDm5; lane 8, bio-TF control; lane 9, mixtures of BL21-pEImC and THP-1 cells. (B) The relative gray intensity analyses of bio-TF-binding ability of seven BL21 strains. The relative gray intensities were calculated as the TF signals relative to the LexA signal. (C and D) Intracellular survival assays of BL21-pEImC and BL21-pEImC-EFG strains in THP-1 cells with or without holo-TF supplementation. Total CFU numbers of bacteria per THP-1 cell were calculated at 0, 1, 2, 4, and 6 h after holo-TF supplementation or not. (E and F) Intracellular survival assays of seven BL21 strains containing a series of pEImC plasmids in holo-TF-supplemented THP-1 cells. Total CFU numbers of bacteria per THP-1 cell were calculated at 0 h and 1 h after holo-TF supplementation. Data in panels C and D are expressed as the mean ± standard deviation. Data in panels B, E, and F are expressed as the mean ± standard error. Statistical differences for comparisons among three or more groups were determined using one-way ANOVA tests and those between two groups were determined using unpaired *t* test. *, *P *<* *0.05; **, *P *<* *0.01; ***, *P *<* *0.001; ns, no significance.

To determine the effects of the interaction between EFG and holo-TF on bacterial viability in macrophages, the ability of BL21 strains containing a series of pEImC plasmids strains to bind holo-TF and their ability to survive in THP-1 cells were determined. As shown in Fig. 9A, the bands corresponding to bio-TF indicated that intracellular bacteria bound to bio-TF. Positive bands for LexA and the negative band for human cellular GAPDH excluded contamination by holo-TF in THP-1. The relative gray intensity of the bio-TF band with respect to the LexA band of seven BL21 strains containing a series of pEImC plasmids is showed in Fig. 9B. These results indicated that BL21-pEImC-EFG and BL21-pEImC-ΔDm1 to BL21-pEImC-ΔDm5 had a significantly stronger ability to bind intracellular TF than the BL21-pEImC strain (*P *<* *0.05). However, the deletion of Dm1 and Dm2 impaired the holo-TF-binding ability of BL21-pEImC-EFG (*P *<* *0.05).

In intracellular survival assays, the total CFU numbers of BL21-pEImC and BL21-pEImC-EFG per THP-1 cell with or without holo-TF are plotted in Fig. 9C and D and Fig. S6. The supplementation of holo-TF in macrophages significantly increased the number of bacteria in each cell for both the BL21-pEImC-EFG and BL21-pEImC strains (*P *<* *0.005), which suggested that holo-TF promoted the tolerance of bacteria to macrophages. Furthermore, the survival ability of BL21-pEImC-EFG was significantly stronger than that of the BL21-pEImC strain in holo-TF-supplemented THP-1 cells (*P *<* *0.001) (Fig. 9D). No significant difference was observed in the survival ability of BL21-pEImC and BL21-pEImC-EFG or BL21-pEImC-ΔDm1 to BL21-pEImC-ΔDm5 in macrophages without the addition of holo-TF (*P > *0.05) (Fig. 9E), which excluded the influence of different strains on THP-1 cells. The deletion of any of the domains significantly decreased the ability of BL21-pEImC-EFG to survive in holo-TF-added macrophages (*P *<* *0.05) (Fig. 9F). These results indicated that the binding of EFG and holo-TF promoted the survival of E. coli BL21 in macrophages. The deletion of EFG Dm1 and Dm2 reduced the ability of E. coli to bind holo-TF in macrophages. Furthermore, the deletion of any domain of EFG impaired the survival ability of bacteria in THP-1 cells.

## DISCUSSION

Metal ions (e.g., iron, manganese, zinc, and copper) are the components of metalloproteins, including metalloenzymes, transcription factors, and storage proteins (30). These ions play an important role in diverse fundamental processes (31). Moreover, these metal ions are essential nutrients required for the viability of microorganisms and host cells (32). To prevent bacterial infection, the host restricts the bioavailability of metal ions to bacteria. This process is termed nutritional immunity, which constitutes a line of defense for the host innate immune system (33).

Plundering iron from the host is determinantal to the pathogenicity of bacteria. Bacteria have evolved iron acquisition mechanisms to circumvent the iron sequestering mechanisms of host, including siderophore-based acquisition systems, heme acquisition systems, and TF/lactoferrin acquisition systems (34). Siderophores are iron chelators secreted by bacteria that bind ferric iron with extraordinary affinity (35). The siderophore-iron complex is bound by receptors present on the surface of the bacterium. Iron is released after it is internalized into bacteria (36). For example, the *Enterobacteriaceae* enterobactin has been shown to chelate and subsequently import iron through FepA receptors in the FepBCDG system (37). The heme acquisition system utilizes surface receptors to bind heme or hemoproteins. Heme is internalized into the cytoplasm and is degraded by heme-catabolizing enzymes (38). For example, the presence of PhuR surface expression on Pseudomonas aeruginosa can bind heme and then transfer it into the cytoplasm via the PhuT-PhuUV system (39). In addition, the TF/lactoferrin acquisition system utilizes surface receptors to recruit TF/lactoferrin and remove iron from them. Iron is transported to the cytoplasm through the FbpABC system, such as the TbpA/TbpB described in the introduction (10).

Although there have been several studies on the iron acquisition mechanism of Gram-negative bacteria, research specific to E. coli remained to be fully elucidated. E. coli can synthesize siderophores (e.g., enterobactin and salmochelin) to acquire ferric iron or secrete hemolysin to release hemoglobin-bound heme from erythrocytes (40). Uropathogenic E. coli recruits Rab35, a protein involved in the TF receptor cycle, to acquire iron (41). Enteropathogenic E. coli binds to TF via OmpA and OmpC. Although studies have shown that E. coli can directly bind to TF, as well as indirectly use the TF-associated iron, there have been few studies on E. coli that directly use the iron in TF (20). Our study found that holo-TF was directly bound and utilized by ExPEC RS218 to increase both its growth in culture medium and its survival in macrophages.

In M9 culture medium, the addition of holo-TF was able to accelerate the growth of RS218. However, the same number of bacteria reached the plateau phase, which was due to the limitation of other nutrients in the M9 medium, including carbon and nitrogen sources. In addition, the addition of low-dosage (20 μg/mL) holo-TF had a stronger effect on bacterial growth than the high-dosage holo-TF (50 μg/mL). This is because excessive iron is harmful to bacteria. Moreover, iron is a mediator of oxidative stress. Excess iron can produce hydroxyl free radicals that damage DNA and proteins, which affects bacterial growth (42, 43).

To study the mechanism by which ExPEC RS218 uses holo-TF, the combination of RS218 and holo-TF was tested. Some bacteria, such as uropathogenic E. coli, obtain iron from the labile iron pool in host cell, although some of the iron in the unstable iron pool comes from holo-TF (41). However, some bacteria, such as M. tuberculosis, directly combine with intracellular holo-TF to obtain iron (19). The observation of the colocalization of RS218 and holo-TF in macrophages suggested a novel mechanism for E. coli to acquire intracellular iron. Furthermore, it also suggested that the competitive binding of holo-TF between RS218 and cells was one of the intracellular survival mechanisms of ExPEC. The binding of holo-TF to RS218 in a liquid environment and in THP-1 cells also indicated the presence of TF-binding proteins. Although TbpA and TbpB are representative TF-binding proteins, the BLAST analysis (https://blast.ncbi.nlm.nih.gov/Blast.cgi) of the E. coli genome did not identify TbpA-like or TbpB-like proteins. To identify the holo-TF-binding proteins associated with ExPEC RS218, desthiobiotin pulldown assays were used to capture the bound membrane proteins with bio-TF. Surprisingly, EFG was screened as a holo-TF-binding protein.

In general, EFG is a cytoplasmic protein. In this study, the surface location of EFG was confirmed. In various pathogens, EFG, elongation factor Tu, and glycolytic enzymes without signal peptides are detected in membrane components (44–46). These proteins are known moonlighting proteins, which are secreted to bacteria surface via certain nonclassical secretion pathways and play a role in the binding host proteins (47). EFG is typically a GTPase involved in protein synthesis (48). In addition, EFG is the target of the antibiotic fusidic acid, mediating the resistance of bacteria to fusidic acid (49–51). The novel moonlighting role of EFG bound with holo-TF was revealed in this study. The holo-TF-binding regions were also explored. Recombinant proteins that lack some EFG regions had a stronger holo-TF-binding capacity than the full-length EFG, which suggested that these proteins expose more binding sites. The TF-related iron-releasing function of EFG and its domain was also confirmed. In this study, we found that the binding of EFG to holo-TF and the release of iron after binding were implemented by different domains of EFG, which acted cooperatively in iron acquisition. EFG is a conserved protein in various pathogens with more than 55% sequence identity (Fig. S7A). The conservation of EFG is as high as 100% among various E. coli isolates, such as other pathotypes, commensal strains, or even laboratory workhorse strains (Fig. S7B). It is speculated that EFG might have the potential to bind TF and release iron from TF if EFG is a surface protein in these E. coli strains even in these various pathogens.

Research into the EFG of ExPEC and GAPDH of M. tuberculosis has suggested that for bacteria that do not possess professional TF-binding proteins, the moonlighting protein is a key breakthrough in studying the mechanism of iron uptake in holo-TF. Furthermore, we also found that although both EFG and GAPDH were moonlighting proteins with the function of binding holo-TF, their iron uptake mechanisms, especially iron release mechanisms, were quite different.

In Gram-negative bacteria, iron from siderophores, heme, and TF requires the TonB-ExbB-ExbD system for transportation across the outer membrane to the cytoplasm (33). These siderophore receptors, heme receptors, and TF receptors that rely on the TonB-ExbB-ExbD system are termed TBDRs. These TBDRs interact with the C terminus of TonB (25). Previous studies have shown that TBDR ligands can enhance the interaction between TBDR and TonB (52). However, no interaction between EFG and TonB was detected, regardless of the existence of holo-TF (data not shown). This result indicated that the EFG was not a TBDR. In addition, when SWISS-MODEL (https://swissmodel.expasy.org/) was used to predict the EFG structure, the result presented in Fig. S8 indicated that the EFG did not contain a 22-stranded β-barrel or an N-terminal plug domain, which are common structures for most TBDRs. This result further confirms that EFG was not a TBDR. Previous studies have also reported that TbpA and TbpB utilize FbpA rather than TonB to mediate iron transport (26). Moreover, a BLAST analysis of the E. coli genome did not find FbpA-like genes. Thus, the specific transport mechanism by which iron is released by EFG requires further research.

As an essential gene of E. coli, the *efg* knockout strain was not utilized in this study (53). To study the function of EFG at the bacterial level, a surface expression vector was constructed. There was no significant difference in the level of mCherry expression on different BL21 containing a series pEImC plasmids, which eliminated the influence of mCherry or InaZN on the results of subsequent experiments. The surface expression of EFG did not affect the expression of OmpA or that of cytoplasmic EFG, indicating that the vector used in this study did not affect the expression of other membrane or cytoplasmic proteins. By using these vectors, it was further confirmed that the surface overexpression of EFG promoted the binding of bacteria to holo-TF, iron uptake, and intracellular survival. The effects of different EFG domains were also verified at the whole bacterium level. The effect of EFG in promoting intracellular survival of E. coli suggested that EFG is an important virulence factor.

Our findings reveal a novel mechanism of TF-iron acquisition in ExPEC. Moreover, ExPEC exhibited surface EFG expression to specifically recruit holo-TF in both a liquid environment and macrophages. As a novel holo-TF-binding protein, EFG could both distinguish holo-TF from apo-TF and release the iron in holo-TF from bacterial surface. The holo-TF binding and iron release functions of EFG are coordinated by different domains, among which the N-terminal domains possess the holo-TF-binding ability, whereas the C-terminal domains possess the iron release ability. Therefore, ExPEC utilizes EFG to acquire holo-TF in macrophages and promote its survival ability. This study provides novel avenues for future research into the molecular mechanisms of ExPEC resistance to innate immunity.

## MATERIALS AND METHODS

### Strains, plasmids, and cell lines.

The bacterial strains and plasmids used in this study are listed in Table 1. ExPEC strain RS218 (O18:K1) is a prototypic meningitis strain isolated from the cerebrospinal fluid of a neonate (23). Unless otherwise indicated, the bacteria were cultured in Luria broth medium. When antibiotics were required, kanamycin and ampicillin were added at 50 μg/mL and 100 μg/mL, respectively. THP-1 cells (a human macrophage cell line) were cultured in RPMI 1640 medium with 10% fetal bovine serum and induced with phorbol 12-myristate 13-acetate as described previously (1).

### ExPEC RS218 growth kinetics.

RS218 was cultured to the logarithmic phase. The collected bacteria were washed with iron-free M9 minimal culture medium. Bacteria were reinoculated into a 96-well plate in 200 μL M9 medium supplemented with 0, 5, 10, 20, and 50 μg/mL holo-TF (Sigma, USA). Bacteria were cultured at 37°C with shaking at 180 rpm in a Tecan Spark microplate reader. The value of OD_600_ was measured each hour.

### ExPEC RS218 TF-binding assays.

TEM assays were performed as previously described with few modifications (19). Log phase RS218 were washed twice with PBS containing 0.2% casein followed by blocking with PBS containing 2% casein at for 1 h 4°C. The bacteria were incubated with colloidal gold-conjugated holo-TF, apo-TF (Sigma, USA), and CA (Meek, Germany) in PBS containing 0.2% casein, respectively, for 2 h at 4°C. The colloidal gold-conjugated holo-TF, apo-TF, and CA were prepared as described previously (54, 55). Colloidal gold was prepared in our laboratory. Bacteria were fixed with 2.5% glutaraldehyde. After washing with 5 mM NaCl, the bacteria were viewed using TEM (HITACHI HT7800).

Fresh ExPEC strain RS218 (2.0 × 10^8^ CFU) was blocked as described in TEM assays. The bacteria were incubated with 20 μg fluor-TF alone or in the presence of unlabeled holo-TF for 1 h at 4°C. Bacteria were incubated with unlabeled holo-TF functioned as the background control. Bacteria with FITC-apo-TF were also subjected to the same process. The fluor-TF was purchased from Life Technologies (USA). FITC-apo-TF was prepared by labeling apo-TF with FITC (Thermo Fisher Scientific, USA) according to manufacturer’s instructions. After washing, the fluorescence intensity of the 10^7^ cells was detected at an excitation of 485 nm and an emission of 535 nm. The fluorescence of the background control group was subtracted from all values. The ExPEC incubated with fluor-TF was observed under a fluorescence microscope.

### Separation of bacterial fractions.

Total membrane proteins, outer membrane proteins, and cytosolic fractions of E. coli were isolated as described previously (1, 21). To isolate cytosolic proteins, bacteria were lysed by sonication at 4°C. The lysate was centrifuged at 10,000 × *g* for 10 min at 4°C and then at 30,000 × *g* for 1 h at 4°C. The supernatant obtained was further centrifuged at 100,000 × *g* for 2 h at 4°C. Finally, the supernatant contained the cytosolic fraction, which was verified by Western blotting using anti-LexA antibodies (Abcam, USA). Anti-OmpA antibodies prepared in our laboratory (1) were used to verify that the cytoplasmic fractions did not contain membrane proteins.

To isolate total membrane proteins, bacteria were washed with PBS, resuspended in PBS with 1.0% Triton X-114, and incubated for 4 h at 4°C. The supernatant was collected by centrifugation at 10,000 × *g* for 10 min, subjected to phase separation at 37°C for 15 min, and centrifuged at 10,000 × *g* for 15 min at 25°C. The detergent phase contained bacterial total membrane fractions. To isolate outer membrane proteins, bacteria were incubated with EZ-Link sulfo-NHS-LC-desthiobiotin, a reagent that cannot penetrate the cell membrane, on ice for 30 min to label outer membrane proteins. Bacteria were washed with 500 mM glycine-PBS to quench excessive desthiobiotin regents. Then, total membrane proteins were extracted as described above. Labeled outer membrane proteins were isolated from membrane fractions with streptavidin agarose resin. The extracted total membrane proteins or outer membrane proteins were verified using anti-OmpA antibodies. Anti-LexA antibodies were used to determine whether the membrane proteins contaminated cytoplasmic proteins.

### Detection of holo-TF internalization.

Log phase ExPEC strain RS218 (5 × 10^9^ CFU) was washed with M9 medium. Then, bacteria incubated with 200 μg bio-TF for 4 h at 37°C or 4°C. Bio-TF was prepared by labeling holo-TF with EZ-Link sulfo-NHS-LC-desthiobiotin (Thermo Fisher Scientific, USA) according to the manufacturer’s instructions. Bacteria were washed four times with cold PBS. The cytosolic fractions were isolated and then incubated with streptavidin agarose resins (Thermo Fisher Scientific, USA). Captured proteins were loaded on a PVDF membrane and detected using horseradish peroxidase (HRP)-streptavidin (Invitrogen, USA). The isolated cytosolic fractions were verified by Western blotting using anti-LexA and anti-OmpA antibodies. The outer membrane proteins of ExPEC RS218 were used as a positive control for anti-OmpA antibodies.

### Expression and purification of recombinant proteins and antibodies preparation.

The primers used in this study are listed in Table 2. The *efg* (locus_tag W817_18790) and its domain deletion genes (ΔDm1 to ΔDm5) or regions deletion genes (Δ4.1 to Δ4.4) were recombined into pET-28a using a ClonExpress II one step cloning kit (Vazyme, China) or ClonExpress MultiS one step cloning kit (Vazyme, China). Recombinant plasmids were transformed into E. coli BL21(DE3). Expression and purification of recombinant proteins were performed as described previously (56).

**TABLE 2:** Primers used in this study

Primer	Oligonucleotide sequence (5′–3′)^*a*^	Product
EFG-F	TGGACAGCAAATGGGTCGCGGATCCATGGCTCGTACAACACCCAT	*efg*
EFG-R	GAGTGCGGCCGCAAGCTTGTCGACGTTATTTACCACGGGCTTCAA	
ΔDm1-F	ATGGGTCGCGGATCCGAATTCATGCCGGTTGACGTACCTGC	ΔDm1
ΔDm1-R	GTGGTGGTGGTGGTGCTCGAGTTATTTACCACGGGCTTCAATTAC	
ΔDm2-F1	ATGGGTCGCGGATCCGAATTCATGGCTCGTACAACACCCATCG	ΔDm2-1
ΔDm2-R1	ATCCGGGTCAACAAACGGGTCGGTAGCG	
ΔDm2-F2	ACCCGTTTGTTGACCCGGATGCGCCGATC	ΔDm2-2
ΔDm2-R2	GTGGTGGTGGTGGTGCTCGAGTTATTTACCACGGGCTTCAATTAC	
ΔDm3-F1	ATGGGTCGCGGATCCGAATTCATGGCTCGTACAACACCCATCG	ΔDm3-1
ΔDm3-R1	AACCTGCGGTTCCATACGTTCCAGAATGATCG	
ΔDm3-F2	AACGTATGGAACCGCAGGTTGCTTACCGTG	ΔDm3-2
ΔDm3-R2	GTGGTGGTGGTGGTGCTCGAGTTATTTACCACGGGCTTCAATTAC	
ΔDm4-F1	ATGGGTCGCGGATCCGAATTCATGGCTCGTACAACACCCATCG	ΔDm4-1
ΔDm4-R1	GCAGAACTGGTTTACCTACGTTCGCTTCAACG	
ΔDm4-F2	CGTAGGTAAACCAGTTCTGCTTGAGCCGAT	ΔDm4-2
ΔDm4-R2	GTGGTGGTGGTGGTGCTCGAGTTATTTACCACGGGCTTCAATTAC	
ΔDm5-F	ATGGGTCGCGGATCCGAATTCATGGCTCGTACAACACCCATCG	ΔDm5
ΔDm5-R	GTGGTGGTGGTGGTGCTCGAGTTATGGTTTAGCTTTCTTAAAGC	
Δ4.1-F	ATGGGTCGCGGATCCGAATTCATGTCTGGTATGGCTAAGCAGTATG	Δ4.1
Δ4.1-R	GTGGTGGTGGTGGTGCTCGAGTTATTTACCACGGGCTTCAATTAC	
Δ4.2-F1	ATGGGTCGCGGATCCGAATTCATGGCTCGTACAACACCCATCG	Δ4.2-1
Δ4.2-R1	CGGTCCATTTTCCAGAATGCAGTAGTCGCAGC	
Δ4.2-F2	GCATTCTGGAAAATGGACCGCATGGGTG	Δ4.2-2
Δ4.2-R2	GTGGTGGTGGTGGTGCTCGAGTTATTTACCACGGGCTTCAATTAC	
Δ4.3-F1	ATGGGTCGCGGATCCGAATTCATGGCTCGTACAACACCCATCG	Δ4.3-1
Δ4.3-R1	GCTCTTCAGAAGCGTTAACGAACGCAATACGCGG	
Δ4.3-F2	CGTTAACGCTTCTGAAGAGCTGATGGAAAA	Δ4.3-2
Δ4.3-R2	GTGGTGGTGGTGGTGCTCGAGTTATTTACCACGGGCTTCAATTAC	
Δ4.4-F1	ATGGGTCGCGGATCCGAATTCATGGCTCGTACAACACCCATCG	Δ4.4-1
Δ4.4-R1	TTCAGCTGCGGATTCGATCA	
Δ4.4-F2	TGATCGAATCCGCAGCTGAACCGGTTGACGTACCTGCG	Δ4.4-2
Δ4.4-R2	GTGGTGGTGGTGGTGCTCGAGTTATTTACCACGGGCTTCAATTAC	
EFG-pEImC-F	GGTTCTGGATCCGAATTCGAGCTCATGGCTCGTACAACACCCATCGCAC	EFG-pEImC
EFG-pEImC-R	TGGTGGTGGTGGTGGTGCTCGAGTTTATTTACCACGGGCTTCAATTACG	
ΔDm1-pEImC-F	GGTTCTGGATCCGAATTCGAGCTCATGCCGGTTGACGTACCTGC	ΔDm1-pEImC
ΔDm1-pEImC-R	TGGTGGTGGTGGTGGTGCTCGAGTTTATTTACCACGGGCTTCAATTAC	
ΔDm2/3/4-pEImC-F	GGTTCTGGATCCGAATTCGAGCTCATGGCTCGTACAACACCCATCG	ΔDm2/3/4-pEImC
ΔDm2/3/4-pEImC-R	TGGTGGTGGTGGTGGTGCTCGAGTTTATTTACCACGGGCTTCAATTAC	
ΔDm5-pEImC-F	GGTTCTGGATCCGAATTCGAGCTCATGGCTCGTACAACACCCATCG	ΔDm5-pEImC
ΔDm5-pEImC-R	TGGTGGTGGTGGTGGTGCTCGAGTTTATGGTTTAGCTTTCTTAAAGC	

a Underlined sequences of primers correspond to restriction enzyme recognition sites. GTCGAC, *Sal* I; GGATCC, *BamH* I; GAATTC, *EcoR* I; CTCGAG, *Xho* I; GAGCTC, *Sac* I.

The rabbit anti-EFG antibodies were prepared by Shanghai Willget Biotechnology Co., Ltd. The peptide used to prepare antibodies had the sequence SFRVWTDEESNQT. To determine the specificity of EFG antibodies, Western blotting was performed. Target protein band was detected with Western ECL substrate (Bio-Rad, USA) and the Bio-Rad ChemiDoc touch imaging system (Bio-Rad, USA) according to the manufacturer’s instructions.

### Far-Western blotting.

The far-Western blotting was performed as previously described with modifications (57). The holo-TF, rEFG, and rHis proteins were subjected to SDS-PAGE and transferred to PVDF membranes. After blocking with 2% bovine serum albumin (BSA), the PVDF membrane was incubated with 10 μg/mL holo-TF or rEFG for 2 h. After washing, PVDF membranes incubated with holo-TF were incubated with rabbit anti-TF antibodies and PVDF membranes incubated with rEFG were incubated with anti-EFG antibodies at 4°C for 4 h. The membranes were washed and then incubated with HRP-conjugated goat anti-rabbit IgG antibodies (Abcam, USA).

### ELISA plate binding assays.

The ELISA plate binding assays were performed as previously described with modifications (1, 58). Recombinant EFG or its proteins with domains or regions deleted and rHis (0, 0.01, 0.1, 0.2, and 0.5 μM) were immobilized in ELISA plates. The wells were blocked with 2% BSA. The interaction was detected by a sequential incubation with 0.2 μM bio-TF and HRP-streptavidin for 2 h at 4°C. The interaction between rEFG and bio-apo-TF was also detected as described above. The preparation method of bio-apo-TF was consistent with the preparation method of bio-TF.

### Recombinant protein inhibition assays.

Fluor-TF (20 μg) was incubated with rEFG (0, 5, 10, and 20 μg/mL) for 2 h at 4°C. The mixtures were coincubated with ExPEC RS218 as described in the holo-TF-binding assays of ExPEC. Bacteria incubated with fluor-TF were used as the reference control. The fluorescence intensity of 10^7^ bacterial cells was detected. Data were calculated as the fold change relative to the reference group.

### Desthiobiotin pulldown assays of holo-TF with rEFG and ExPEC total membrane proteins.

Desthiobiotin pulldown assays were performed according to the manufacturer’s instructions of an EZ-Link desthiobiotinylation and pulldown kit (Thermo Fisher Scientific, USA). Bio-TF (50 μg) and desthiobiotin labeled (bio-CA, 50 μg) were incubated with streptavidin agarose resins according to the manufacturer’s instructions. Bio-CA was prepared as described in the bio-TF preparation section. The interacted proteins were RS218 total membrane proteins (1 mg) or rEFG (100 μg). Captured proteins were detected by Western blotting using anti-EFG antibodies. To detect the total membrane fractions in this assay, Western blotting was performed using anti-OmpA and anti-LexA antibodies, and the whole bacterial proteins were used as a control.

### Holo-TF conversion detection of recombinant proteins.

The rEFG (0, 0.1, 0.2, 0.5, 1 μM) and ΔDm1 to ΔDm5 (1 μM) were incubated with bio-TF (0.1 μM) for 2 h in a total volume of 200 μL PBS at 4°C or 37°C. The same concentration of bio-TF was used as a reference. The protein mixtures were diluted and coated on an ELISA plate. The OD_450_ values of iron-carrying TF and total TF signals in the mixtures were detected using anti-TF antibodies and HRP-streptavidin, respectively. Background readings of the wells coated without proteins were subtracted from the samples. The relative OD_450_ value was calculated as the ratio of the OD_450_ value of the iron-carrying TF signal to that of total TF signal.

### Detection of the EFG surface location.

Western blotting, colony blotting, and suspension immunofluorescence assays were performed as previously described with some modifications (1, 59). Outer membrane proteins and cytoplasmic proteins (negative control) of RS218 were separated and detected by Western blotting. For colony blotting, ExPEC outer membrane proteins were stained on the nitrocellulose membrane through contact with bacterial colonies. For immunofluorescence detection, whole bacteria were incubated with antibodies. Anti-EFG, anti-OmpA, anti-LexA, and HRP-anti-rabbit IgG antibodies were used for Western blotting and colony blotting. In addition to the above primary antibodies, mouse anti-RS218 antiserum, FITC-conjugated goat anti-mouse IgG, and TRITC-conjugated goat anti-rabbit IgG antibodies were used for immunofluorescence detection.

### Heterologous protein surface-displaying strain construction and verification.

The heterologous protein surface-display plasmid was constructed as described in previous studies with some modifications (27–29). Genes encoding InaZN and mCherry fused with the GGGGS linker in the 5′ and 3′ regions of *mCherry* were recombined into the pET-28a vector. All of the above genes were codon optimized and synthesized by GenScript (Nanjing). The new plasmid was termed pEImC. *efg* and ΔDm1 to ΔDm5 were recombined into the pEImC plasmid, respectively. The recombinant plasmids were termed pEImC::*efg*, pEImC::ΔDm1, pEImC::ΔDm2, pEImC::ΔDm3, pEImC::ΔDm4, and pEImC::ΔDm5. All seven of these series pEImC plasmids were transformed into E. coli BL21(DE3).

BL21-pEImC-EFG, BL21-pEImC-ΔDm1 to BL21-pEImC-ΔDm5, and BL21-pEImC were induced by 0.5 mM IPTG for 4 h at 37°C. Next, the induced whole bacteria were separated by SDS-PAGE. To detect the mCherry fluorescence, the induced BL21-pEImC-EFG strain was observed under a fluorescence microscope. We measured the mCherry fluorescence intensity of 1 × 10^7^ cells of the seven induced BL21 strains containing a series of pEImC plasmids at an excitation of 579 nm and an emission of 624 nm. Background readings of uninduced bacterial controls were subtracted from the samples. The surface expression of EFG in the BL21-pEImC-EFG and BL21-pEImC strains was measured by whole strain ELISAs as described previously (1). The anti-EFG antibodies and HRP-goat anti-rabbit IgG antibodies were used in this assay. The outer membrane proteins and cytoplasmic proteins of the BL21-pEImC-EFG and BL21-pEImC strains were subjected to Western blotting. The anti-EFG and anti-OmpA antibodies were used to detect the outer membrane proteins. The anti-EFG and anti-LexA antibodies were used to detect the cytoplasmic proteins. The gray intensity of the band was analyzed in Image J software.

### Holo-TF-binding assays of BL21 strains containing a series of pEImC plasmids.

The holo-TF-binding assays of the BL21-pEImC-EFG and BL21-pEImC strains were performed as described in the ExPEC RS218 TF-binding assays section with some modifications, for which the concentration of fluor-TF was 20 μg/mL. For BL21-pEImC-ΔDm1 to BL21-pEImC-ΔDm5, bacteria were incubated with fluor-TF alone. The fluorescence intensity of fluor-TF on the surfaces of these seven BL21 strains containing a series of pEImC plasmids was detected.

### Calcein-AM fluorescence quenching assays.

Calcein-AM fluorescence quenching assays were performed as described previously with modifications (19, 60). Log phase ExPEC strain RS218 and seven BL21 strains containing a series of pEImC plasmids were washed twice with M9 medium. Then, 1 × 10^8^ bacterial cells were incubated with 1 μM calcein-AM (Sigma, USA) for 150 min at 37°C. After washing, the bacteria were incubated with or without holo-TF for 6 h at 37°C. For the ExPEC, BL21-pEImC-EFG, and BL21-pEImC strains, the concentration of supplementary holo-TF was 0, 5, 10, 20, and 50 μg/mL. For the BL21-pEImC-ΔDm1 to BL21-pEImC-ΔDm5 strains, the holo-TF concentration was 10 μg/mL. After washing, the fluorescence intensity of 10^7^ cells was measured at an excitation of 490 nm and an emission of 538 nm.

### Conversion detection of holo-TF.

Fresh ExPEC strain RS218 was washed twice with M9 medium. Then, 1 × 10^8^ bacterial cells were incubated with bio-TF (0, 5, 10, 20, and 50 μg/mL) in M9 medium for 6 h at 37°C or 4°C. The bacteria were then placed on ice for 10 min to stop the iron acquisition process. Culture supernatants were diluted and coated on an ELISA plate. The total TF and iron-carrying TF signals in the culture supernatants were detected using HRP-streptavidin and anti-TF antibodies, respectively. The value of the blank control M9 medium was subtracted from all values. The same concentration of bio-TF was used as the reference control. The relative values of total TF signals or iron-carrying TF signals were calculated as the ratio of the OD_450_ value of the experimental group to the OD_450_ value of the reference group.

For seven BL21 strains containing a series of pEImC plasmids, bacteria were incubated with 10 μg/mL bio-TF at 37°C. OD_450_ values of total TF and iron-carrying TF signals in culture supernatants were detected as described above. The relative values were calculated as the ratio of the OD_450_ value of the iron-carrying TF signal to the OD_450_ value of the total TF signal.

### Intracellular survival assays.

Intracellular survival assays were performed according to previous studies with some modifications (21). ExPEC strain RS218 and seven BL21 strains containing a series of pEImC plasmids were incubated with THP-1 cells for a multiplicity of infection (MOI) of 10. After incubation for 1 h, cells were washed with PBS. Extracellular bacteria were killed by gentamicin for 1 h. Cells were washed, and different dosages of holo-TF were added to cells. For ExPEC strain RS218, the dosages of holo-TF were 0, 5, 10, 20, and 50 μg/mL. For the seven BL21 strains, the dosage of holo-TF was 20 μg/mL. The number of cells and bacteria in the cells before holo-TF supplementation (0 h) and after holo-TF supplementation (1, 2, 4, and 6 h) was measured. The live cell count was performed as described previously (61). After the cells were washed three times with PBS, 0.25% trypsin (400 μL) was added to digest cells. Cell suspensions (10 μL) were incubated with 0.4% trypan blue (10 μL, Thermo Fisher Scientific), and then cell numbers were detected with a Countess instrument (Thermo Fisher Scientific). For the counts of intracellular bacteria, 600 μL of PBS containing 0.1% Triton X-100 was added to cell suspensions to release intracellular bacteria. Bacterial numbers were measured by plate counting. Total CFU numbers of bacteria per cell at 0, 1, 2, 4, and 6 h time points were calculated as the ratio of the number of bacteria to the number of cells. To determine the intracellular survival ability of the seven BL21 strains containing a series of pEImC plasmids in THP-1, total CFU numbers of bacteria per cell after 0 h and 1 h of holo-TF supplementation were measured.

### Colocalization detection of holo-TF and ExPEC RS218 in macrophages.

THP-1 cells were incubated with RS218 as described in the intracellular survival assay. The extracellular bacteria were cleaned at 1 h postincubation. The cells were incubated with 20 μg fluor-TF for 1 h at 4°C. Subsequently, the cells were shifted to 37°C at 5% CO_2_ for 1 h to allow for fluor-TF internalization. After washing, the cells were fixed, permeabilized, and blocked as described previously (62). The intracellular bacteria were labeled with rabbit anti-RS218 antiserum and TRITC-goat anti-rabbit IgG antibodies. The cells were observed under a fluorescence microscope.

### Detection of the holo-TF-binding ability of intracellular BL21 strains containing a series of pEImC plasmids.

Seven BL21 strains containing a series of pEImC plasmids were incubated with THP-1 cells (∼2 × 10^7^) as described in the colocalization detection assay. After the extracellular bacteria were cleaned, cells were incubated with 1 mg bio-TF for 1 h at 4°C. Then, cells were incubated at 37°C, 5% CO_2_ for another 1 h. Cells were washed four times with cold RPMI 1640 medium. The intracellular E. coli cells were isolated from THP-1 cells as described previously (63). Briefly, THP-1 cells were lysed in 0.1% SDS in the presence of 1,000 units/mL DNase (Invitrogen, USA) and the EDTA-free protease inhibitor mixture tablet (Thermo Scientific Pierce, USA) to release intracellular bacteria. The lysate was centrifuged at 4°C for 10,000 × *g* to remove the supernatant. Bacteria were washed three times with 0.01% SDS in RPMI 1640. Finally, the bacteria were resuspended in 0.01% SDS in RPMI 1640 and filtered through a 2 mL, 0.22 μm Spin-X centrifuge tube filter (Costar, USA). The filter containing the captured E. coli was cut and subjected to Western blotting. Bio-TF, bacteria, and THP-1 cells were used as controls. Anti-LexA antibodies were used to detect bacteria recovered from inside THP-1 cells. Anti-GAPDH antibodies (Abcam, USA) were used to detect the THP-1 cells, and HRP-streptavidin was used to detect the bio-TF bound with bacteria.

### Statistical analysis.

All data were obtained from at least three independent experiments. GraphPad Prism version 8.0.1 was used to analyze data. Data are expressed as the mean ± standard error or mean ± standard deviation. Statistical analyses for multigroup and pairwise comparisons were assessed using one-way analysis of variance (ANOVA) and unpaired *t* test, respectively. The significant difference was accepted as *P *<* *0.05.
