# Supplementary material for: Extraintestinal Pathogenic Escherichia coli Utilizes Surface-Located Elongation Factor G to Acquire Iron from Holo-Transferrin
Source: Microbiol Spectr. 2022 Mar 7;10(2):e01662-21. doi: 10.1128/spectrum.01662-21 (PMC9045202; doi:10.1128/spectrum.01662-21)
Supplement: SUPPLEMENTAL FILE 1 — Supplemental material. Download SPECTRUM01662-21_Supp_1_seq14.pdf, PDF file, 4 MB [file spectrum01662-21_supp_1_seq14.pdf]

Supplementary Results

Identification of holo-TF binding proteins of ExPEC RS218

Desthiobiotin pull-down assays were performed from ExPEC RS218 total membrane fractions to screen holo-TF-binding proteins. The pull-down using Bio-CA (EZ-Link sulfo-NHS-LC desthiobiotin labeled CA, a negative control) did not screen any interacting proteins, which indicated that the proteins identified by the desthiobiotin pull-down with Bio-TF were specific (Fig. S3A). A total of 13 protein spots were successfully identified by mass spectrometry. These proteins were considered potential holo-TF binding proteins (Table S1, Fig. S3B).

Supplementary Materials and methods

Desthiobiotin pull-down assays of holo-TF with ExPEC total membrane proteins

Desthiobiotin pull-down assays were performed using an EZ-Link desthiobiotinylation and pull-down kit (Thermo Fisher, USA) according to the manufacturer's instructions. Briefly, Bio-TF and Bio-CA were incubated with streptavidin agarose resins, respectively. After washing, the agarose resins were incubated with ExPEC total membrane fractions for 2 h, then washed five times. The bound proteins were eluted by Biotin Elution Buffer (4 mM biotin, 20 mM Tris, and 50 mM NaCl). To identify holo-TF binding proteins, protein samples were separated by two-dimensional electrophoresis, and protein spots were analyzed by mass spectrometry.

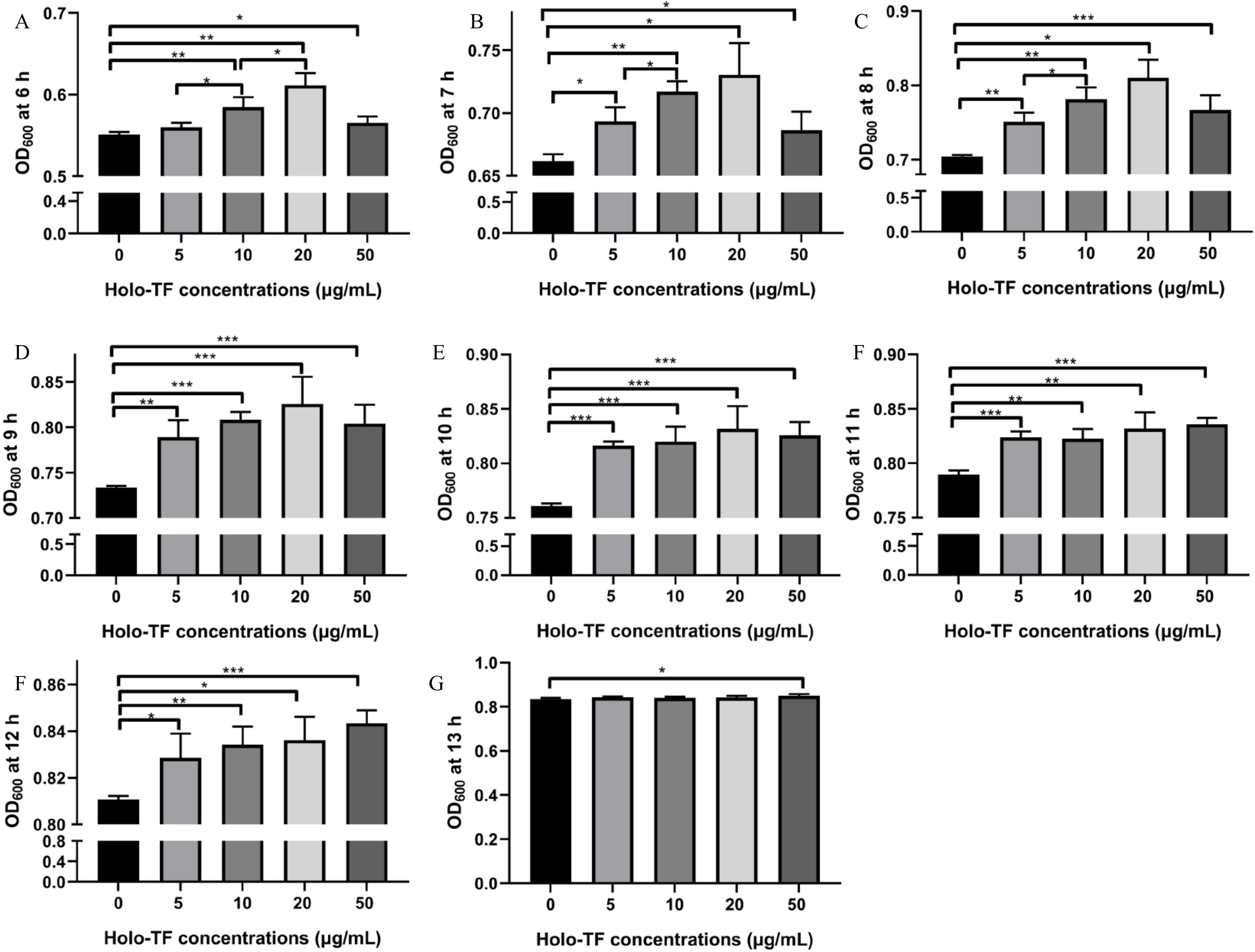

Figure S1. ExPEC strain RS218 was cultured in M9 medium with different concentrations of holo-TF or in the absence of holo-TF. The OD_{600} values were measured each hour. The bar graph displays results of OD_{600} for 6 h to 13 h. Data are expressed as the mean ± standard deviation. Statistical analyses were performed with unpaired *t* test. *, $P < 0.05$. **, $P < 0.01$. ***, $P < 0.001$. ns, no significance.

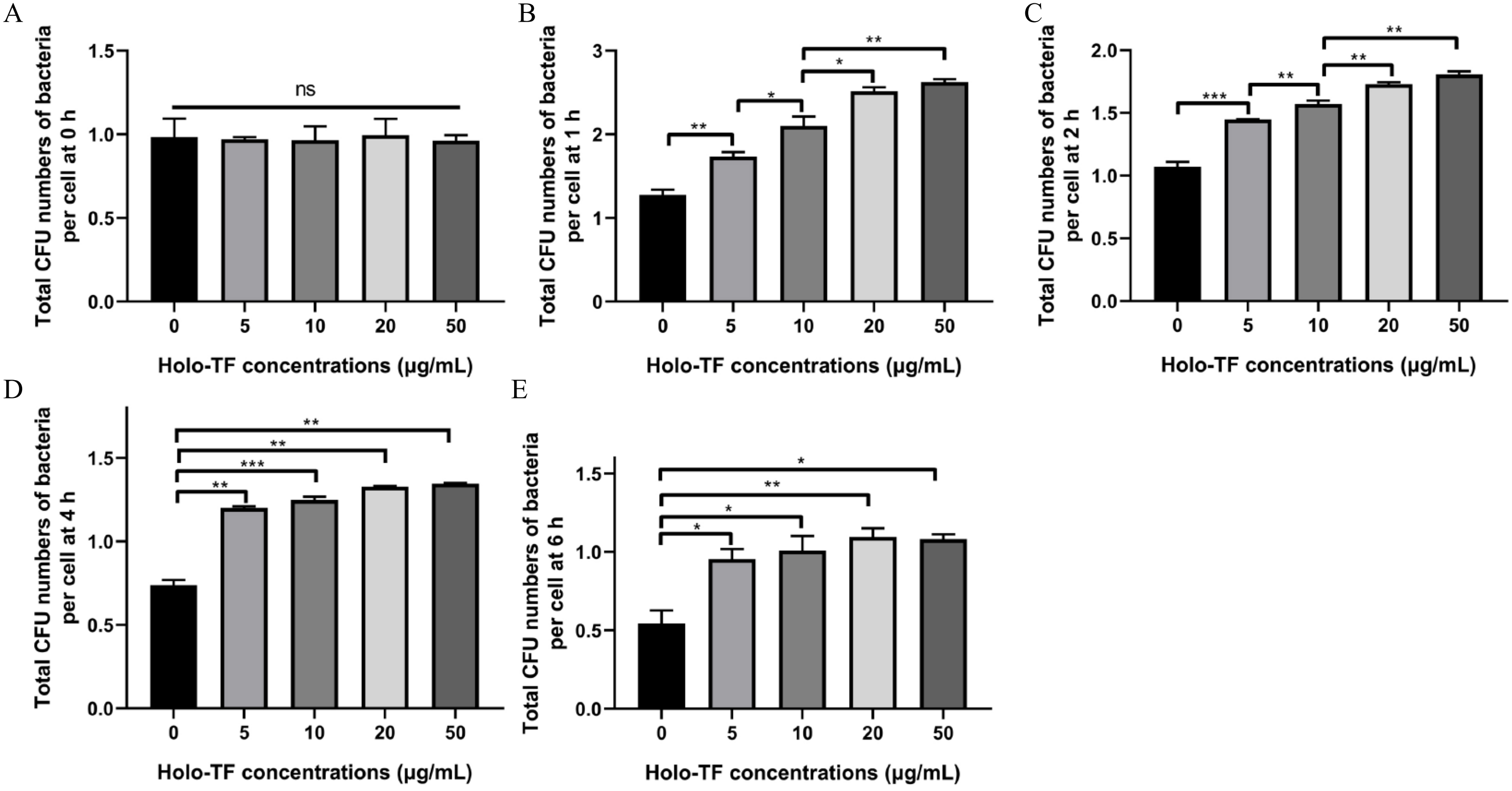

Figure S2. Survival ability of ExPEC RS218 in THP-1 cells with different concentrations of holo-TF. The total numbers of bacteria per THP-1 cell were measured 0, 1, 2, 4, and 6 h after addition of holo-TF. Data are expressed the mean ± standard deviation. Statistical analyses for all pairwise comparisons were assessed using unpaired *t* test. *, $P < 0.05$. **, $P < 0.01$. ***, $P < 0.001$. ns, no significance.

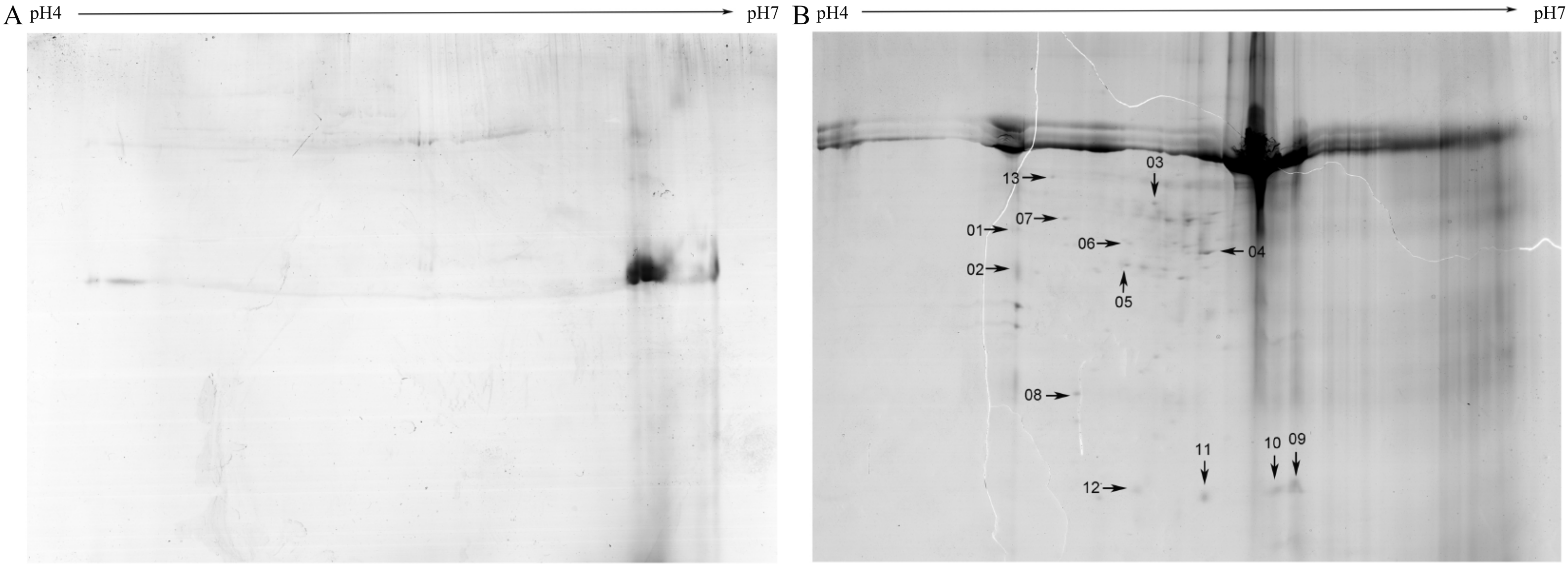

Figure S3. Two-dimensional electrophoresis (2DE) identification of ExPEC membrane proteins interacting with Bio-CA (A) and Bio-TF (B). Arrows on the gel image indicate successfully identified proteins using mass spectrometry.

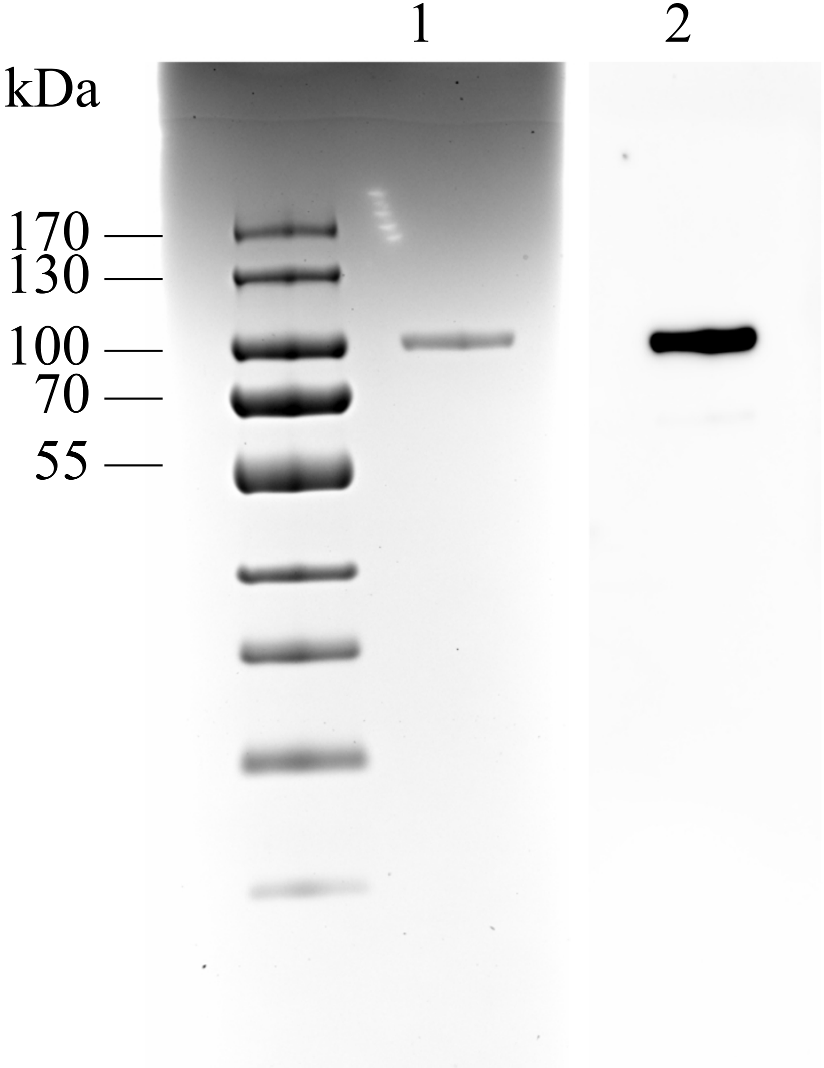

Figure S4. Recombinant expression and antibodies preparation of EFG. The rEFG was subjected to SDS-PAGE and stained with Coomassie G-250 (lane 1). The rEFG was hybridized to anti-EFG antibody and HRP-conjugated anti-rabbit IgG (lane 2).

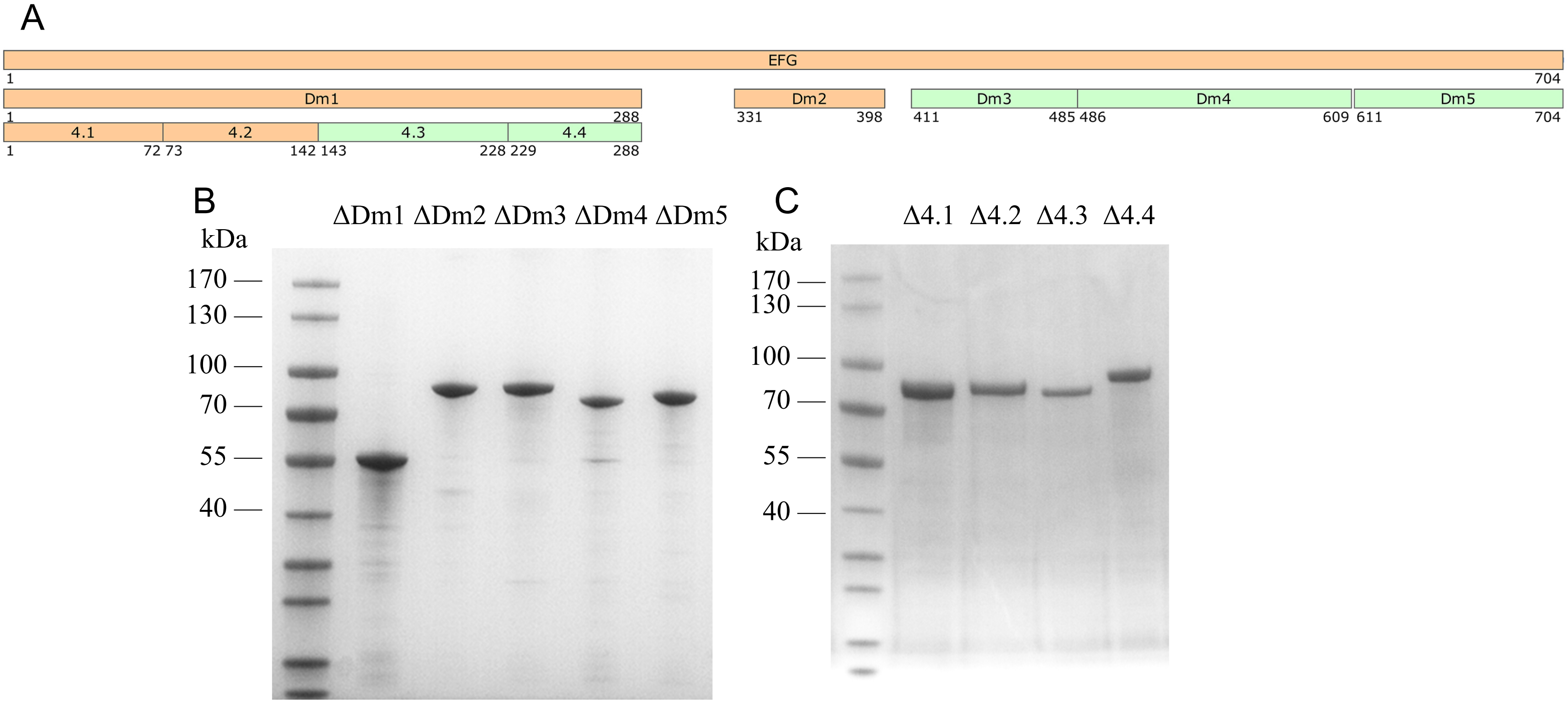

Figure S5. Schematic representation of EFG, Dm1 to Dm5, and 4.1 to 4.4 (A). SDS-PAGE analysis of ΔDm1 to ΔDm5 and Δ4.1 to Δ4.4 (B, C).

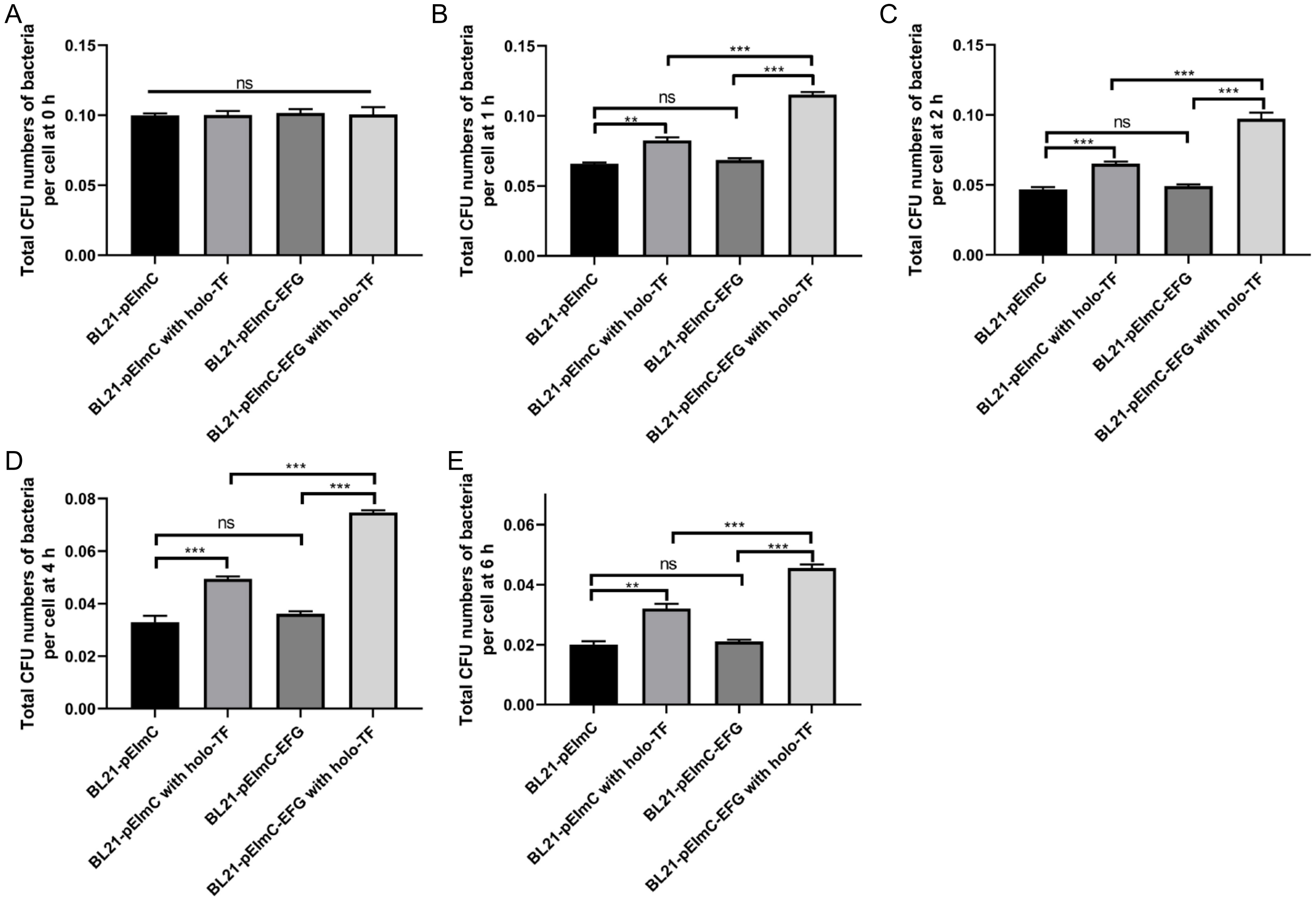

Figure S6. Intracellular survival assays of BL21-pEImC and BL21-pEImC-EFG strains when THP-1 cells were supplemented with or without holo-TF. Total CFU numbers of bacteria per THP-1 cell were calculated at 0, 1, 2, 4, and 6 h after holo-TF supplementation or not. Data are expressed as the mean ± standard deviation. Statistical differences were determined using unpaired *t* test. **, $P < 0.01$. ***, $P < 0.001$. ns, no significance.

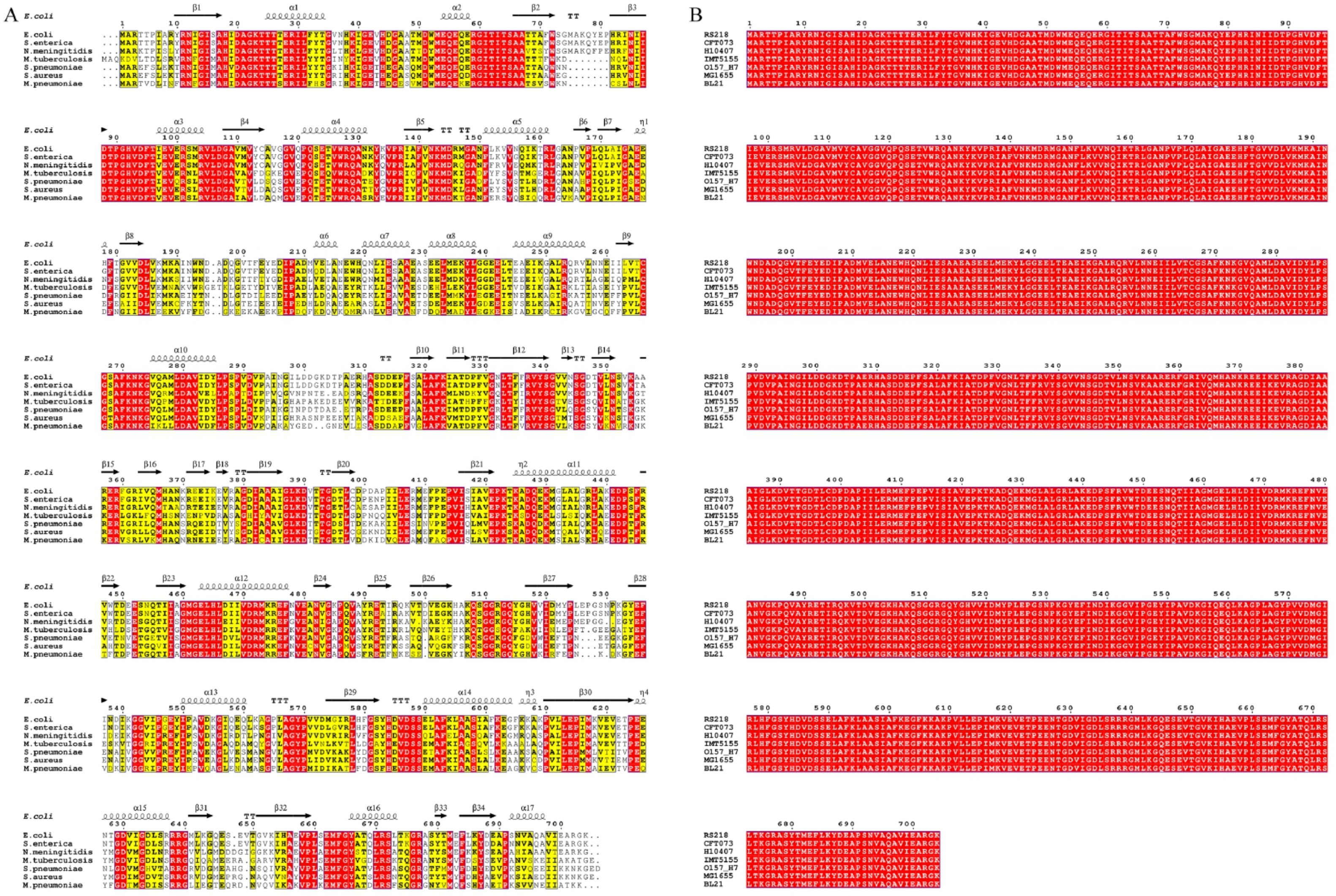

Figure S7 Multiple sequence alignments of EFG from different bacterial pathogens (A) and different *E. coli* strains (B). The multiple sequence alignments were performed by Clustal W. Results rendering were performed by ENDscript/ESPript. *S. enterica*, *Salmonella enterica*. *N. meningitidis*, *Neisseria meningitidis*. *M. tuberculosis*, *Mycobacterium tuberculosis*. *S. pneumoniae*, *Streptococcus pneumoniae*. *S. aureus*, *Staphylococcus aureus*. *M. pneumoniae*, *Mycoplasma hyopneumoniae*. CFT073, an uropathogenic *E. coli* strain. H10407, an enterotoxigenic *E. coli* strain. IMT 5155, an avian pathogenic *E. coli* strain. O157 H7, an enterohemorrhagic *E. coli* strain. MG1655, a commensal *E. coli* strain. BL21, an engineered *E. coli* strain.

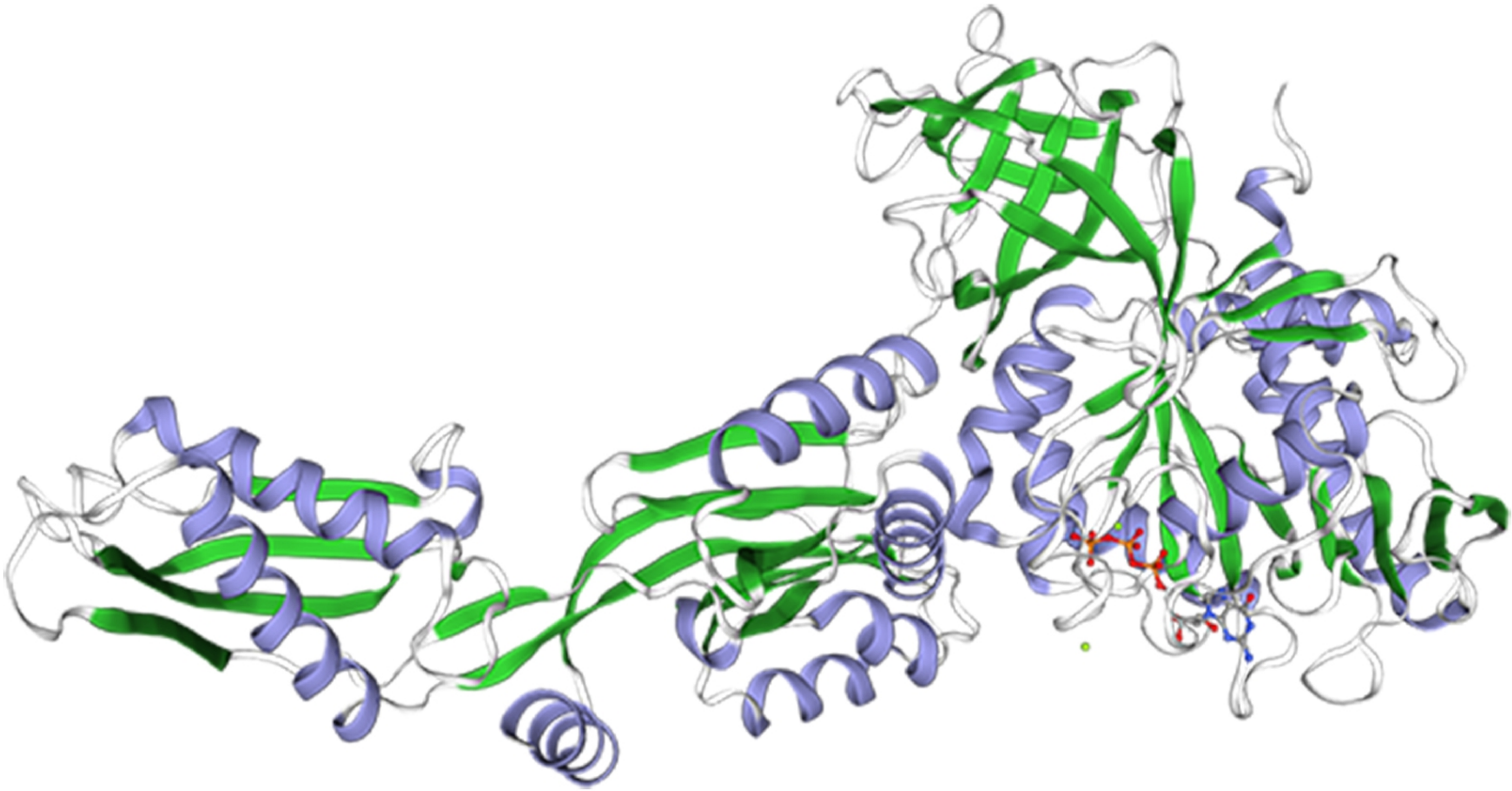

Figure S8 The three-dimensional structure prediction of ExPEC EFG. The structure prediction and visualization were performed by Swiss-model.

Table S1 Identification of holo-TF binding proteins of ExPEC RS218.

Spot No.[a]	Accession No.	Identified protein	Number(s) of matched peptide(s)	Mass[b]	pI[b]
01	AJM73311.1	outer membrane porin protein C	1	39609.57	4.90
02	AJM74404.1	outer membrane porin protein C	1	39995.79	4.58
03	AJM76408.1	aspartate ammonia-lyase	4	52356.13	5.19
04	KIE75509.1	elongation factor Tu	5	43283.55	5.30
05	AJM74998.1	L-1,2-propanediol oxidoreductase	1	40597.49	5.03
06	AJM75539.1	elongation factor G	5	77581.31	5.24
07	AJM75929.1	F0F1 ATP synthase subunit beta	2	50325.42	4.90
08	KIE81268.1	elongation factor P	2	20591.31	4.90
09	AJM73441.1	outer membrane protein W	1	22892.91	6.50
10	AJM73441.1	outer membrane protein W	1	22892.91	6.50

11	AJM74363.1	elongation factor Ts	3	21532.63	4.90
12	AJM75928.1	F0F1 ATP synthase subunit epsilon	3	15068.27	5.46
13	AJM73035.1	hypothetical protein	1	65680.66	4.39

[a]Spot numbers correspond to those indicated in Fig. S3.
[b]Mass and pI were predicted by Compute pI/Mw tool (https://web.expasy.org/compute_pi/).
